# Wavyleaf basketgrass (*Oplismenus undulatifolius*) invasion is associated with changes in soil microbial communities

**DOI:** 10.1128/msphere.00895-25

**Published:** 2026-04-29

**Authors:** Michael R. Fulcher, Anthony Tritz, Vanessa Beauchamp, Carrie A. Wu

**Affiliations:** 1 Foreign Disease-Weed Science Research Unit, Agricultural Research Service, US Department of Agriculture, Frederick, Maryland, USA; 2 Department of Biological Sciences, Towson University 1492, Towson, Maryland, USA; 3 Department of Biology, University of Richmond 6888, Richmond, Virginia, USA; Virginia-Maryland College of Veterinary Medicine, Blacksburg, Virginia, USA

**Keywords:** invasive species, plant-microbe interactions, rhizosphere, soil, microbiome, non-native

## Abstract

Introduced invasive plants can alter the composition of resident soil microbial communities, which may disrupt ecosystem function and facilitate continued invasion success. Wavyleaf basketgrass (*Oplismenus undulatifolius*) is a high-risk, non-native invasive plant currently confined to the Mid-Atlantic United States but with the potential to colonize forest understory across the eastern United States. In this study, we characterized soil microbial communities from locations spanning the invaded range using amplicon sequencing to understand the impacts of wavyleaf basketgrass establishment on resident soil microbiomes. We compared the diversity and structure of microbial communities from invaded and uninvaded forest soils, as well from wavyleaf basketgrass rhizospheres. Invasion by wavyleaf basketgrass was associated with an increase in fungal diversity within sampling locations but a decrease in diversity across sampling locations. Changes in the relative abundance of specific sequence variants indicated a small number of resident microbes may be amplified in wavyleaf basketgrass rhizospheres. Finally, fungal alpha diversity was correlated with soil chemistry variables in uninvaded plots but not in invaded plots, and increased plant ground cover attributed to wavyleaf basketgrass invasion was positively correlated with fungal diversity. Together, these patterns suggest that wavyleaf basketgrass recruits diverse microbial associates from the environment, homogenizes soil microbiomes across invaded locations, and overrides existing environmental selection pressures exerted by soil chemistry profiles. Ongoing expansion of the species’ invaded range may produce similar impacts in new environments.

**IMPORTANCE:**

Understanding whether and how microbial communities are altered by plant invasion provides important information about the impact of introduced species on natural resources, nutrient cycling, and biodiversity that influence subsequent land management and ecosystem restoration decisions. We document biotic homogenization of resident soil microbes across geographically disparate locations following a relatively recent plant invasion. We further provide evidence suggesting microbial community changes are linked to the enrichment of specific taxa from the invasive plant's rhizosphere and possible buffering of these communities against other environmental selective pressures.

## INTRODUCTION

Plant invasions constitute a major form of global environmental change (1) with annual economic impacts estimated in billions of U.S. dollars (2). These invasions are ecological disturbances that can influence plant, animal, and microbial biodiversity, as well as ecosystem function and human health (3–8). Understanding how invasive plants reshape their environment is important to predicting their specific impacts, identifying non-native invasive species management priorities, and potentially informing ecosystem restoration efforts (9–11).

Environmental soil microbiomes, which are vital for plant health and nutrient cycling processes (12), are particularly vulnerable to change caused by invasive plants (13). Non-native plant species have been shown to alter microbial community diversity, structure, and function in a number of ecological contexts (14, 15). These changes can be relatively benign or can, for instance, lead to plant-soil feedbacks that facilitate further invasion success (16–18). Plant invasion impacts on soil microbial communities vary greatly across environments and between different plant taxa (14, 19–22), making it necessary to evaluate these dynamics for individual non-native invasive plant species and habitats as new invasions occur.

Plants can alter soil microbiomes through several distinct mechanisms (23). Plant-directed changes in soil microbial community occur frequently within the rhizosphere, or the area within soil that closely borders root surfaces (24). Plants directly influence soil chemical and structural profiles through root exudation of specific carbon compounds or by larger nutrient releases during tissue decomposition (25). Introduced plants can also provide novel physical niche spaces through varied root architecture (26) and enrich certain microbial taxa through non-random, plant-microbe associations (27). Differentially abundant microbial taxa associated with plant invasion can lead to altered functional diversity in soils (28, 29), and some specifically enriched taxa could alter habitat suitability for future plant communities (30–32).

The non-native, perennial invasive plant wavyleaf basketgrass (*Oplismenus undulatifolius* [Ard.] P. Beauv., Poaceae) is a recent introduction to the United States that currently is restricted to a relatively small invaded range in the Mid-Atlantic region (33). First discovered in the United States in 1996 near Baltimore, MD (34), wavyleaf basketgrass is recognized as a high-risk invasive species by the U.S. Department of Agriculture due to the potential for range expansion across ~30% of the United States (35). This perennial rhizomatous grass forms dense carpets in the forest understory that may crowd out native herbaceous plants and inhibit the regeneration of native hardwood trees (33, 36). Continued wavyleaf basketgrass migration to new forest understory environments has been facilitated by long-distance dispersal of seeds (37). Flowering spikelets with long awns produce an extremely sticky substance that strongly adheres to animals and other objects that brush past the inflorescence (33), and seeds also may contribute to a persistent seed bank (37). Wavyleaf basketgrass continues to spread across the U.S. Mid-Atlantic region, with infestations confirmed in six states as well as the District of Columbia (38).

Infestations of wavyleaf basketgrass are characterized by dense vegetative cover and low native plant diversity (33). Removal of wavyleaf basketgrass infestations by hand-pulling or herbicide application has increased plant species richness, suggesting that the invasion suppresses native plant diversity (36, 39). Similarly, wavyleaf basketgrass impacts the abundance and composition of certain insect taxa where it has invaded, although the specific mechanism underlying this is not known (40). While recent studies have found that soil microbial communities in the Mid-Atlantic region respond to invasion by other herbaceous plant species (41, 42)*,* we do not yet know the impact of wavyleaf basketgrass on local soil microbial communities.

The purpose of this study was (i) to determine whether wavyleaf basketgrass establishment alters soil microbiomes and (ii) to identify conserved microbial associations formed across the plant’s invaded range that will inform future research on the function of invaded soils. This work provides insight into the consequences of wavyleaf basketgrass for resident microbiomes and soil microbial ecology.

## RESULTS AND DISCUSSION

**Fig 1:**
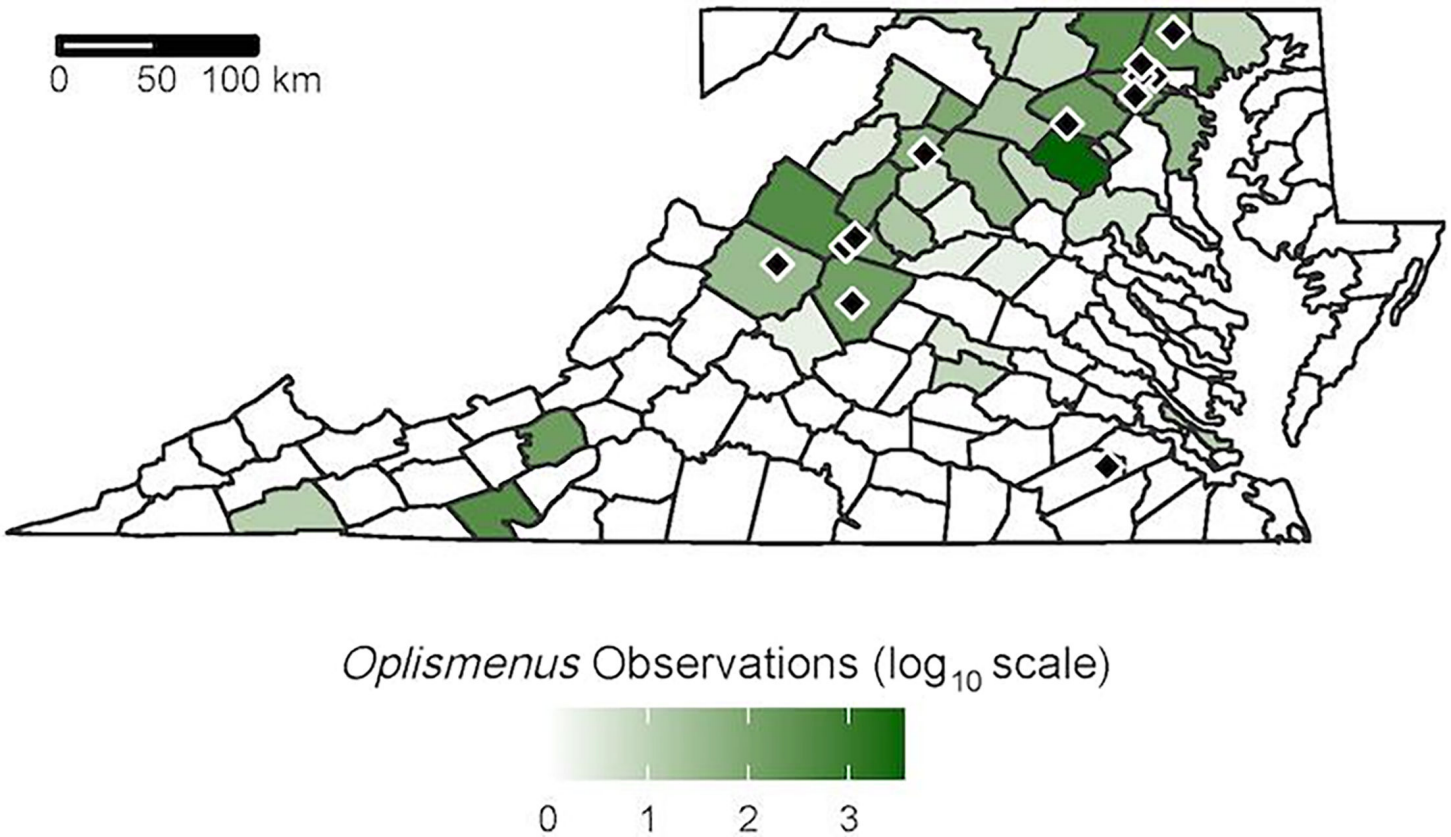
Sampling locations within current *Oplismenus undulatifolius* distribution. Black diamonds mark sample collection locations. County-level *Oplismenus undulatifolius* density is provided based on presence observations from the Early Detection and Distribution Mapping System, University of Georgia, Center for Invasive Species and Ecosystem Health (https://www.eddmaps.org/, accessed 4 October 2023).

Fungal and bacterial soil communities were characterized from wavyleaf basketgrass-invaded and basketgrass-uninvaded forest plots at 12 locations that spanned the core of the introduced range across Maryland and Virginia (Fig. 1). A total of 240 soil samples were collected over 2 years from invaded bulk soil and uninvaded bulk soil. An additional 60 samples of wavyleaf basketgrass rhizosphere soil were collected from 6 invaded locations in the second year of sampling. Amplicon sequencing targeting prokaryotes (16S) and fungi (ITS2) was used to detect differences in microbial communities among these three soil groups. Additionally, soil chemistry and plant community data were analyzed to identify potential drivers of community differentiation between invaded and uninvaded soil microbiomes. This approach captured clear impacts of wavyleaf basketgrass invasion on bulk soil microbiomes, but we acknowledge that conclusions are limited in several key respects. First, the collection of rhizosphere samples was performed in only one of the sampling years, limiting the temporal replication of findings relative to rhizosphere-bulk soil comparisons. Second, while the study captures some level of annual variation by reporting on samples collected over 2 years, no individual plots were represented in both years, preventing analysis of temporal trends. Third, while the observational approach of this work took advantage of the ongoing invasion by wavyleaf basketgrass, experimental manipulation is needed to further understand mechanisms underlying the observed changes.

### Greater local microbial diversity associated with *Oplismenus undulatifolius* invasion may be explained by increases in plant ground cover and buffering against environmental selection

**Fig 2:**
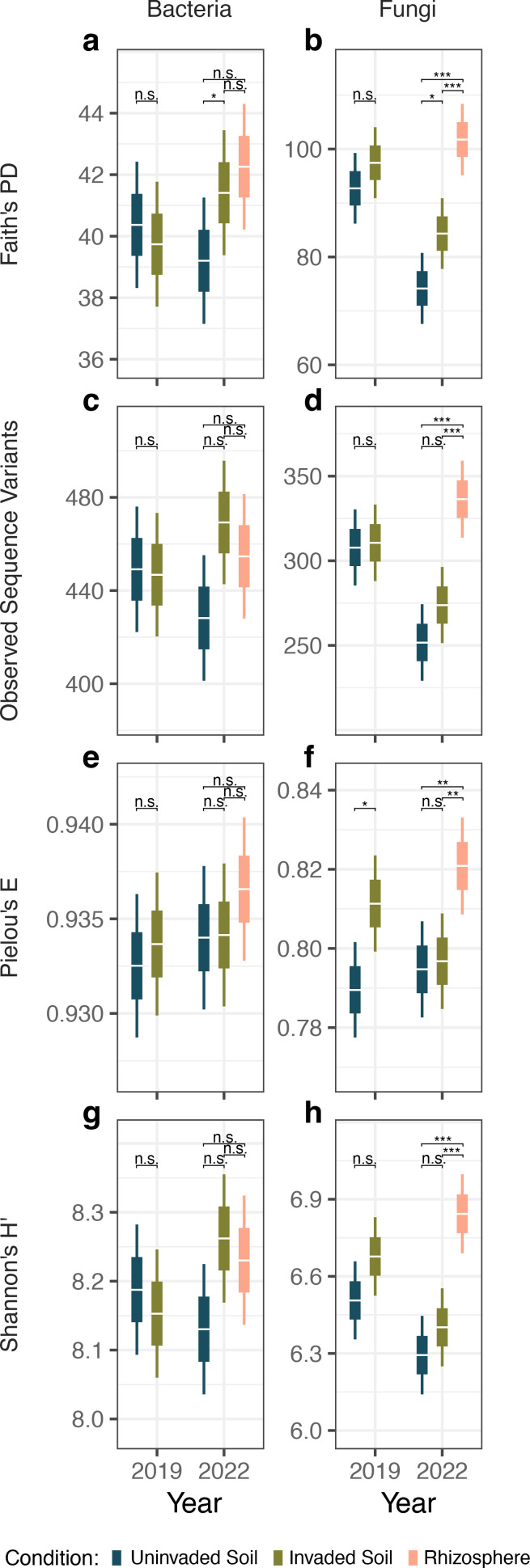
Alpha diversity of 16S and ITS2 sequence variants from uninvaded, invaded, and *Oplismenus undulatifolius* rhizosphere soils. Bacterial and fungal alpha diversity were calculated as Faith’s phylogenetic diversity (**a and b**), the observed number of sequence variants (**c and d**), Pielou’s evenness (**e and f**), and Shannon’s entropy (**g and h**). Horizontal white lines represent mean values, boxes mark standard error, and whiskers show 95% confidence intervals. Each mean and variance measure was calculated using data from 56 to 60 soil samples collected across 6 sampling locations. All calculations and pairwise comparisons were performed within individual sampling years. Brackets and asterisks indicate the significance level of pairwise mean contrast tests with Tukey adjusted *P*-values interpreted as follows: n.s. *P* > 0.05, *P < 0.05, **P < 0.01, ***P < 0.001. Figure shading denotes soil condition.

Wavyleaf basketgrass invasion showed a consistent, positive effect on fungal alpha diversity but not on bacterial alpha diversity in the studied areas based on pairwise contrasts between soils collected within each year of the study (Fig. 2). Soil condition was a significant predictor of fungal alpha diversity in six of eight linear models (Table S1). For all fungal diversity measures, mean diversity was lowest in uninvaded soil and greater in invaded soils. Among samples collected in the second year of study when rhizosphere soils were considered, rhizospheres contained greater fungal diversity than both bulk soil types. Bacterial alpha diversity did not vary significantly between soil conditions, with the exception of phylogenetic diversity in bulk invaded compared to bulk uninvaded soils collected in 2022. Other plant invasions have been associated with increases in bacterial (43, 44) and fungal richness (45–47), though these studies examined only individual microbial kingdoms unlike the present joint analysis. Notably, another invader of Mid-Atlantic forests, the annual grass *Microstegium vimineum*, has been associated with a similar trend of increasing soil bacterial and fungal diversity (41). *Microstegium vimineum* and wavyleaf basketgrass are known to co-occur, and the similar responses of soil microbiome to both invasions could implicate parallel processes of plant-induced environmental change or even a background environment predisposed to experience similar effects from any plant invasion.

**Fig 3:**
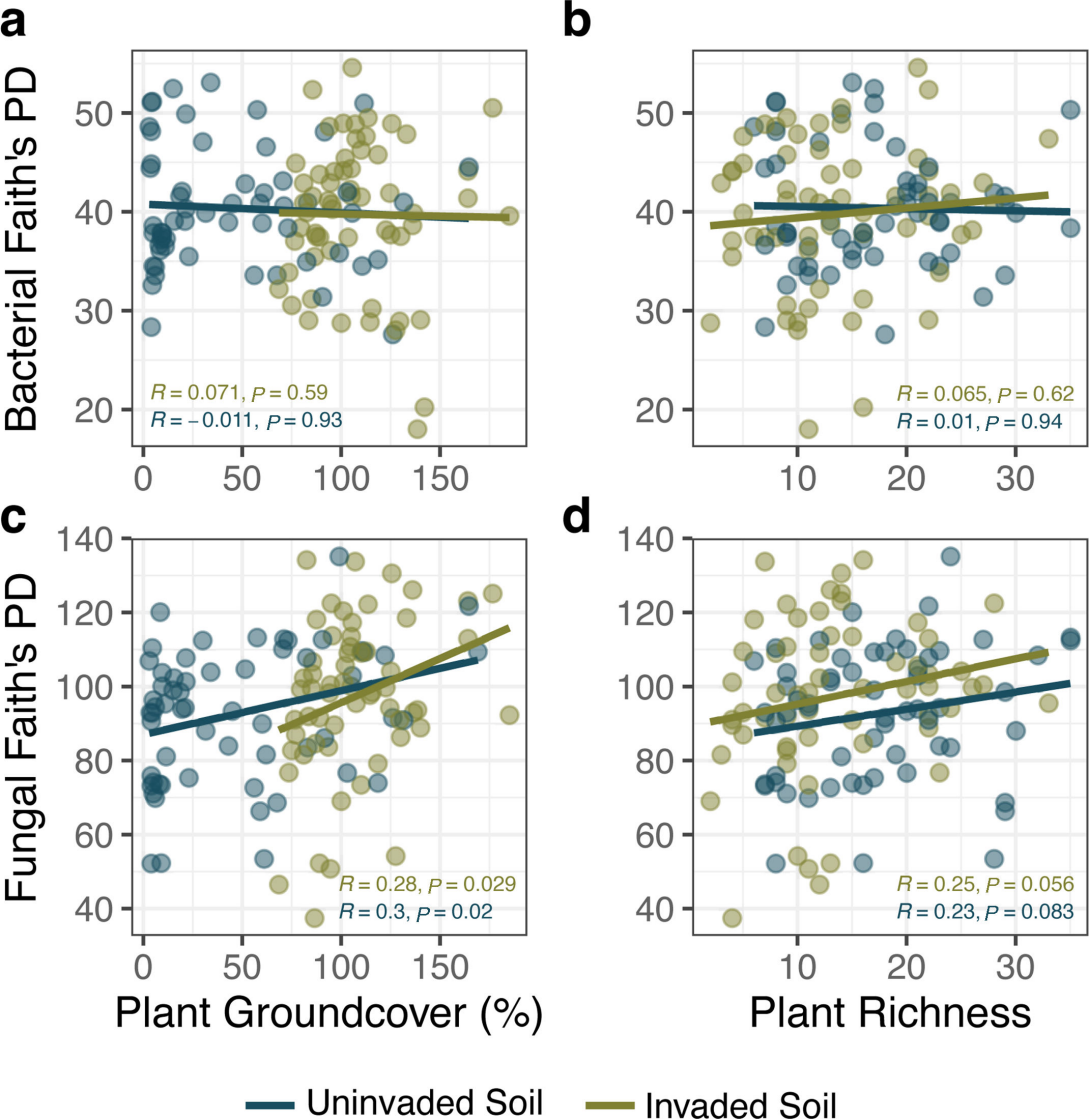
Correlations between microbial sequence diversity and plant community characteristics. Relationships were calculated between Faith’s Phylogenetic Diversity of fungal and bacterial sequences in bulk soils and percent plant groundcover (**a and c**) or plant richness (**b and d**) in soils sampled in 2019 (*n* = 58 to 60 plots per soil condition). Spearman correlation and significance levels are included in each panel, with shading denoting soil condition.

Overall plant ground cover was significantly higher in invaded than uninvaded plots at six of six locations (Kruskal-Wallis *χ*^2^ = 45.245, *P* < 0.001), and plant richness was lower in invaded than uninvaded plots at five of six locations (*P* ≤ 0.008). Fungal phylogenetic diversity had a small positive correlation with plant ground cover but not plant richness, whereas bacterial phylogenetic diversity was not correlated with either measure of plant community (Fig. 3). Above- and below-ground diversity are closely linked, and plant richness has been identified as a driver of soil microbiome diversity (48). However, plant biomass, through correlation with root exudate volume and provision of resources, may be a more important factor for supporting increased microbial abundance (49, 50) and fungal richness (51). Wavyleaf basketgrass invasions have been associated with environments characterized by low-density native plant communities (52), so high-density infestations examined in this study may produce new microclimates or carbon cycling dynamics that favor species-rich fungal communities.

**Fig 4:**
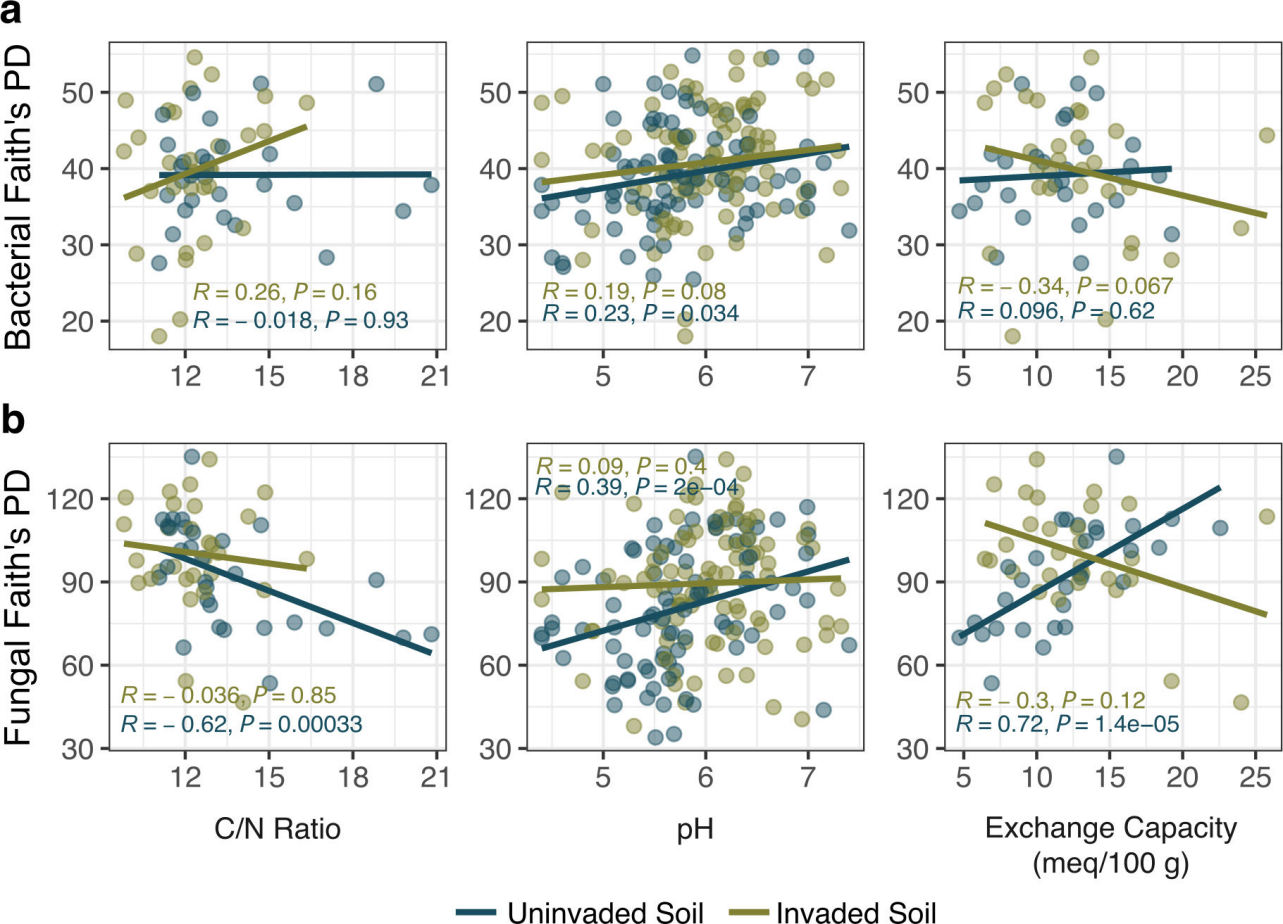
Correlations between microbial sequence diversity and soil chemistry. Relationships were calculated between Faith’s phylogenetic Diversity of bacterial (**a**) or fungal sequences (**b**) in bulk soils and soil C/N ratio, pH, and cation exchange capacity (*n* = 30 soil samples per condition across 6 locations in 2019 for C/N and cation exchange capacity; *n* = 90 soils per condition across 12 total locations from both 2019 and 2022 for pH). Spearman correlation values and significance levels are included in each panel, with shading denoting soil condition.

Wavyleaf basketgrass invasion did not appear to alter soil chemistry profiles. Invaded soils had a higher pH at 4 of 12 locations (Kruskal-Wallis, *P* ≤ 0.021) and a higher C:N ratio at one of six locations (Kruskal-Wallis, *P* = 0.028). There were no differences detected in total cation exchange capacity (CEC) between invaded and uninvaded locations. Fungal diversity had highly significant correlations with all three measures of soil chemistry in uninvaded plots, but these relationships were not detected in invaded plots (Fig. 4). Bacterial diversity could not be explained by soil chemistry metrics, except for a small positive correlation with pH in uninvaded plots.

Soil pH is known to influence microbial communities (53, 54), as are cation exchange capacity (CEC) (55, 56) and C:N ratio (57). Plant invasions can alter soil properties and chemistry (27, 58), but we observed minimal differentiation in soil metrics between wavyleaf basketgrass-invaded and uninvaded plots. We propose instead that wavyleaf basketgrass imposes one or more relatively stronger selective pressures on soil fungal communities, effectively minimizing the role of existing forest soil conditions during microbial community formation at invaded sites. This hypothesis is consistent with the absence of clear changes in the soil properties attributed to wavyleaf basketgrass invasion (39) and the loss of correlation between sequence alpha-diversity and soil chemistry metrics in invaded soils. Wavyleaf basketgrass may also alter the soil environment in ways that override the influence of pH, CEC, and C:N ratio on soil microbiomes. Potential mechanisms for this action include recruitment of diverse microbial phenotypes via specific root exudates and litter nutrient composition, maintenance of consistent soil moisture under dense foliar cover, or increased soil nutrient concentrations that support large, diverse fungal communities. Additional field observations made as the invaded range continues to grow or controlled experiments are needed to test for the influence of these potential mechanisms.

### *Oplismenus undulatifolius* invasion was associated with biotic homogenization of microbial communities

**Fig 5:**
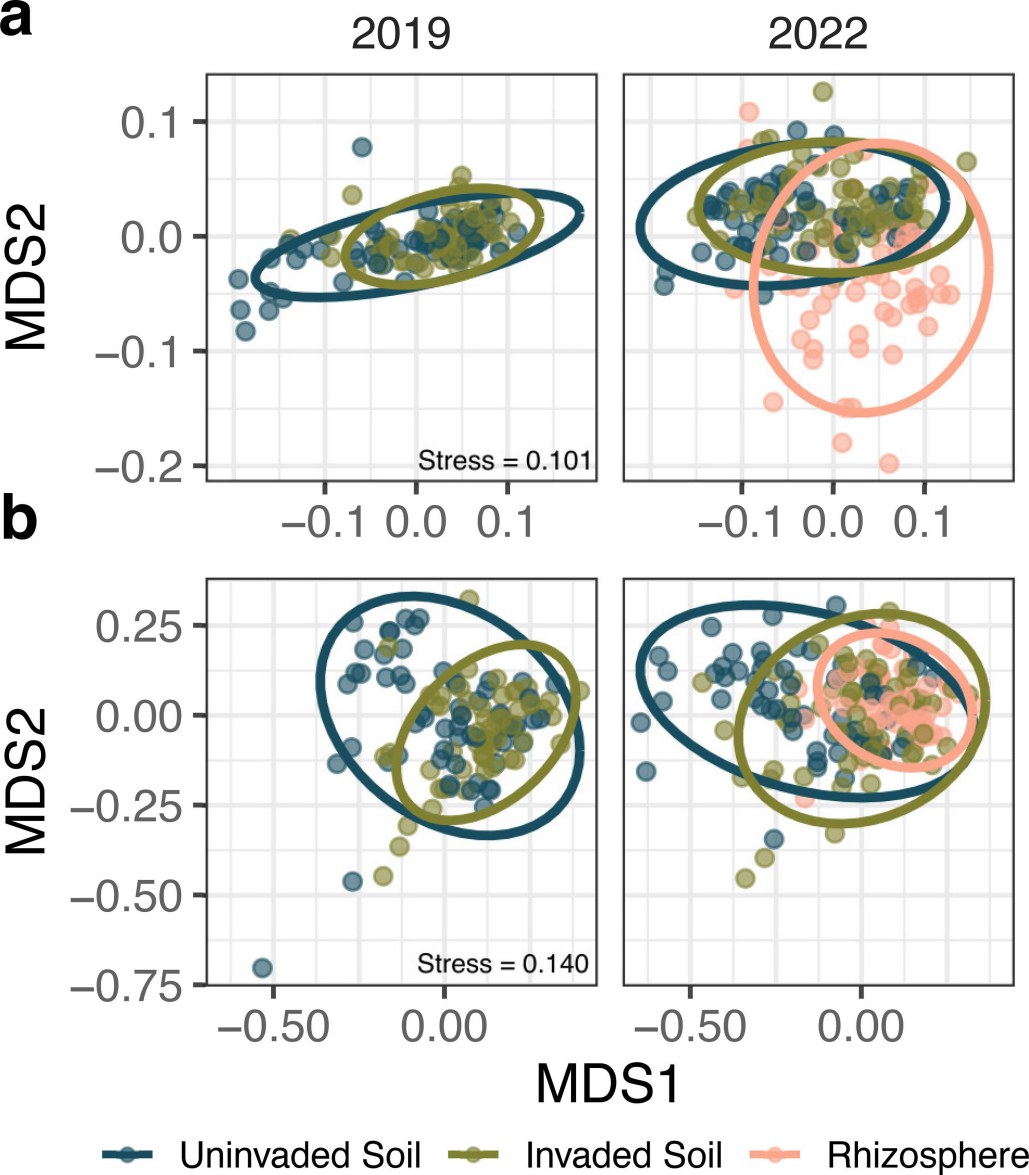
Soil microbial community composition changes with *Oplismenus undulatifolius* invasion. Non-metric multidimensional scaling plots of bacterial (**a**) and fungal (**b**) weighted UniFrac community dissimilarities show differences in the location and dispersion of soil communities. Each point represents a soil sample, shading denotes soil condition, and ellipses mark 95% confidence intervals around condition medians. Plots are divided between samples collected in 2019 and 2022. Each soil condition-by-year combination is represented by 56 to 60 individual soil samples collected across six locations.

The beta diversity patterns observed in wavyleaf basketgrass-invaded and uninvaded soil microbial communities were consistent with expectations for a biotic homogenization process. Biotic homogenization refers to the outcome of several ecological processes that ultimately reduce the regional diversity of communities over time, driving them toward increasingly similar compositions (59). This process is frequently but not exclusively associated with the substitution of non-native, invasive species for previously resident species (60, 61). We used phylogenetically weighted UniFrac distances to assess the beta diversity of soil microbial communities. A nested permutational analysis of variance (PERMANOVA) detected significant differences in weighted UniFrac distances between (i) sample years, (ii) locations sampled within each year, and (iii) soil conditions sampled within each location and year (Table 1). A test of sample dispersion also detected significant differences between soil conditions nested within locations and years for bacteria and fungi (*F*_2,292_ = 6.012, *P* = 0.003; *F*_2,293_ = 5.855, *P* = 0.003). Community similarity was visualized with a non-metric multidimensional scaling (NMDS) plot (Fig. 5), and communities found in bulk invaded soils appeared less dispersed than those found in bulk uninvaded soils.

**TABLE 1:** Nested PERMANOVA comparing microbial communities between *Oplismenus undulatifolius* invaded and uninvaded soils

Parameter	Bacterial communities					Fungal communities				
	d.f.	SS	*R* ^2^	*F*	*P*	d.f.	SS	*R* ^2^	*F*	*P*
Year	1	0.156	0.029	14.647	0.001	1	2.205	0.028	11.507	0.001
Location in year	10	1.421	0.268	13.356	0.001	10	17.844	0.224	8.162	0.001
Condition in location in year	18	0.901	0.170	4.707	0.001	18	11.022	0.138	3.196	0.001
Residual	265	2.818	0.532			266	50.966	0.638		

The increased similarity of microbial communities across locations associated with wavyleaf basketgrass invasion is indicative of a biotic homogenization process spanning the current invaded range, which includes infestations separated by upward of 375 km. Interestingly, a similar loss of beta diversity in soil microbiomes has been associated with invasions of *Spartina alterniflora*, *Solidago canadensis*, and *Vincetoxicum rossicum* in vastly different habitats worldwide (62–64). The homogeneous, high-density plant canopies created by invasive *Alternanthera philoxeroides* have also been theorized to cause a similar pattern of increased microbial richness and decreased beta-diversity in invaded environments (65). The homogenization of soil microbes under high-density plant invasions mirrors the effects observed following other forms of above-ground environmental homogenization, including conversion of forest to pasture (66) and urbanization (67), and thus may present an important mechanism by which invasive plants threaten ecosystem functioning.

### Differences in sequence relative abundance and core microbiomes suggest specific environmental taxa are amplified in rhizospheres during invasion

**Fig 6:**
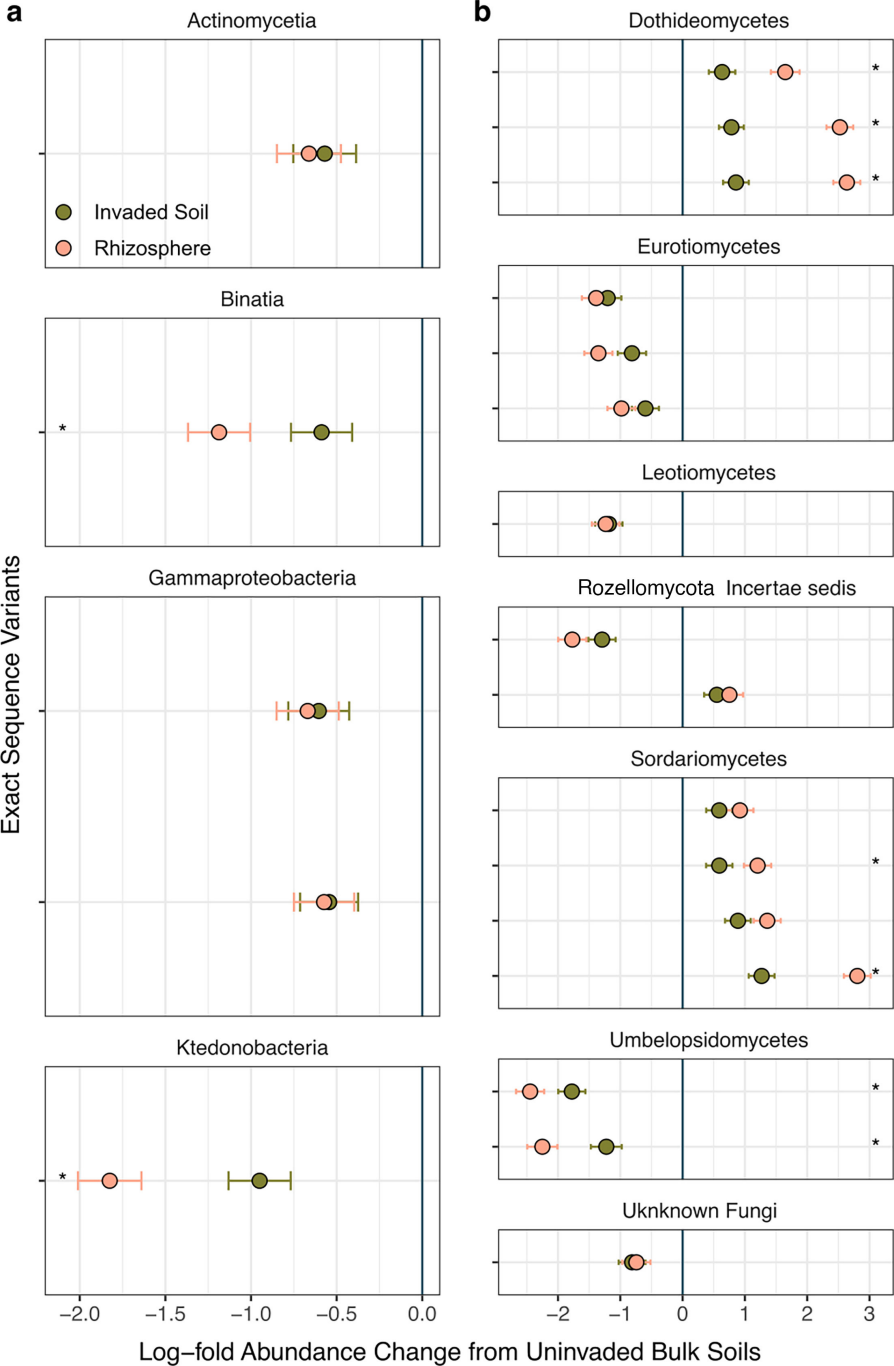
Individual amplicon sequencing variants differed in relative abundance between conditions. A small number of individual bacterial (**a**) and fungal (**b**) sequence variants were found to have significantly different relative abundance in rhizosphere and bulk invaded soils versus uninvaded bulk soils (*P* < 0.05). Sequence variants reported here are based on an analysis of composition of microbiomes (ANCOM-BC2) using only the abundances from samples collected in 2022. Points indicate mean log fold abundance change relative to uninvaded soil, and whiskers represent 95% confidence intervals. Asterisks indicate sequence variants for which rhizosphere and invaded bulk soil abundances also differed significantly from each other in log fold change (*P* < 0.05). Sequence variants are aggregated into panels for different taxonomic classes. Shading denotes soil condition.

An analysis of microbial community composition with bias correction (ANCOM-BC2) detected significant differences in the relative abundance of individual 16S and ITS2 sequence variants at locations where all three soil conditions were sampled in 2022 (68). Five bacterial sequence variants placed in 4 taxonomic classes and 16 fungal sequence variants placed in 7 classes were identified as differing in relative abundance between invaded and uninvaded soils through this analysis (*P* < 0.05) (Fig. 6). For 9 of those 23 total sequences, shifts in relative abundance observed in rhizosphere samples were significantly greater than those observed in bulk invaded soils. That invaded bulk soils typically contained sequence abundance changes intermediate to uninvaded and rhizosphere samples suggests wavyleaf basketgrass root exudates or structures may be responsible for the enrichment of these sequences under invaded conditions. Additional studies are needed to further explore possible mechanisms underlying these changes in microbial relative abundance.

**Fig 7:**
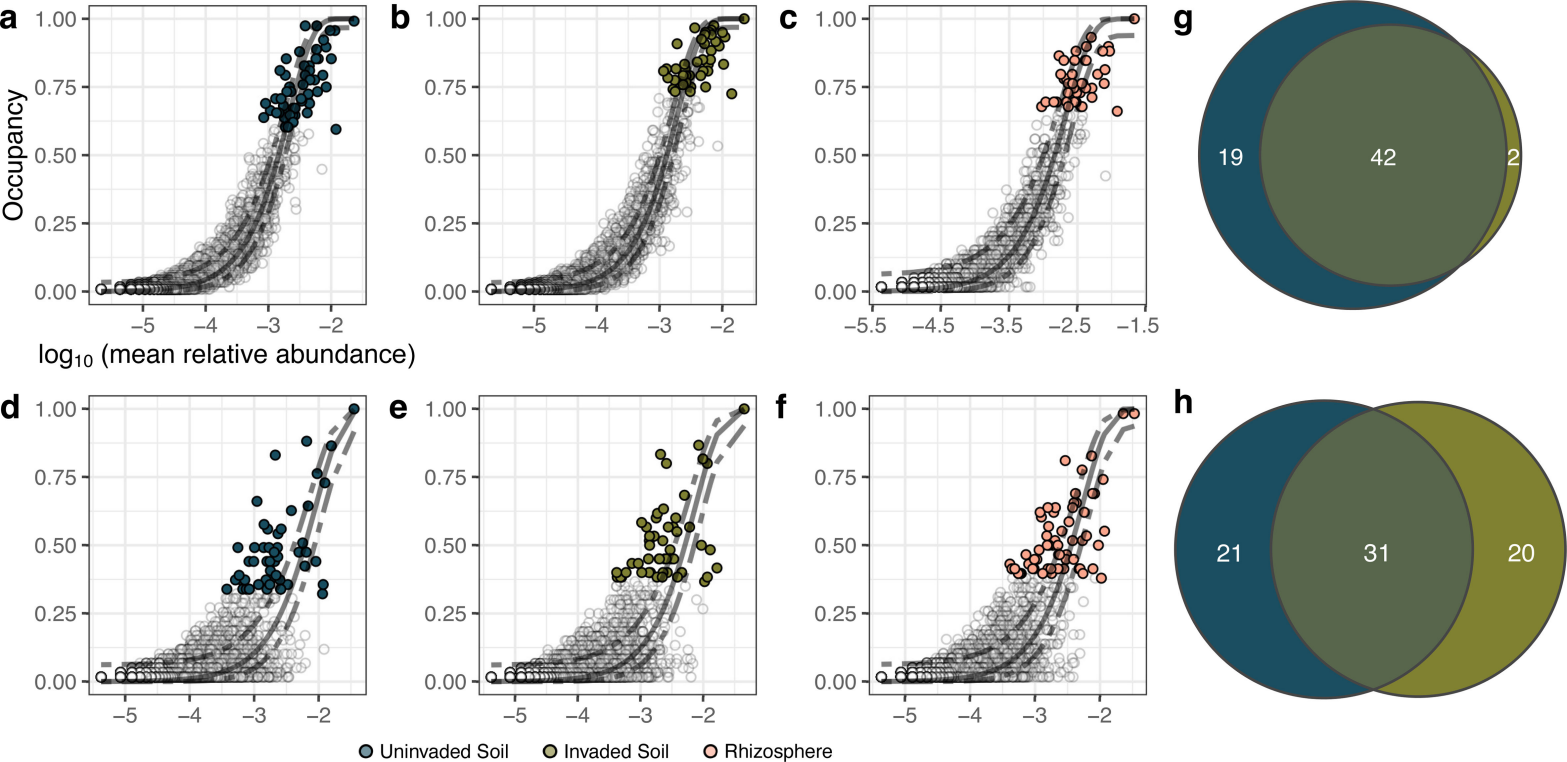
Occupancy-abundance relationships of sequence variants from different soil conditions. Each point represents a unique bacterial (**a–c**) or fungal (**d–f**) sequence variant. Relative abundance and sample occupancy were calculated for bulk uninvaded (**a and d**), bulk invaded (**b and e**), or rhizosphere (**c and f**) soils (fungal *n* = 119 uninvaded and invaded soil samples across 12 locations and 2 years, *n* = 58 rhizosphere samples across 6 locations in 2022; bacterial *n* = 116 uninvaded soils and 120 invaded soils across 6 locations and 2 years, *n* = 59 rhizosphere soil samples across 6 locations in 2022). Shaded points represent sequence variants’ assigned membership to the core microbiota as defined by their contribution of >5% Bray-Curtis community dissimilarity among soil samples. In each panel, the solid curve represents a prediction for the relationship between occupancy and abundance based on a neutral model using only a migration (dispersal) estimate, and dashed lines mark two standard deviations above and below this prediction. Sequence variants placed above the upper dashed line are interpreted to be under selection by their environment, while those placed under the lower line are interpreted to be dispersal limited. Panels g and f show the relative overlap of shared versus unique sequences assigned to core membership in the bulk soil samples.

**Fig 8:**
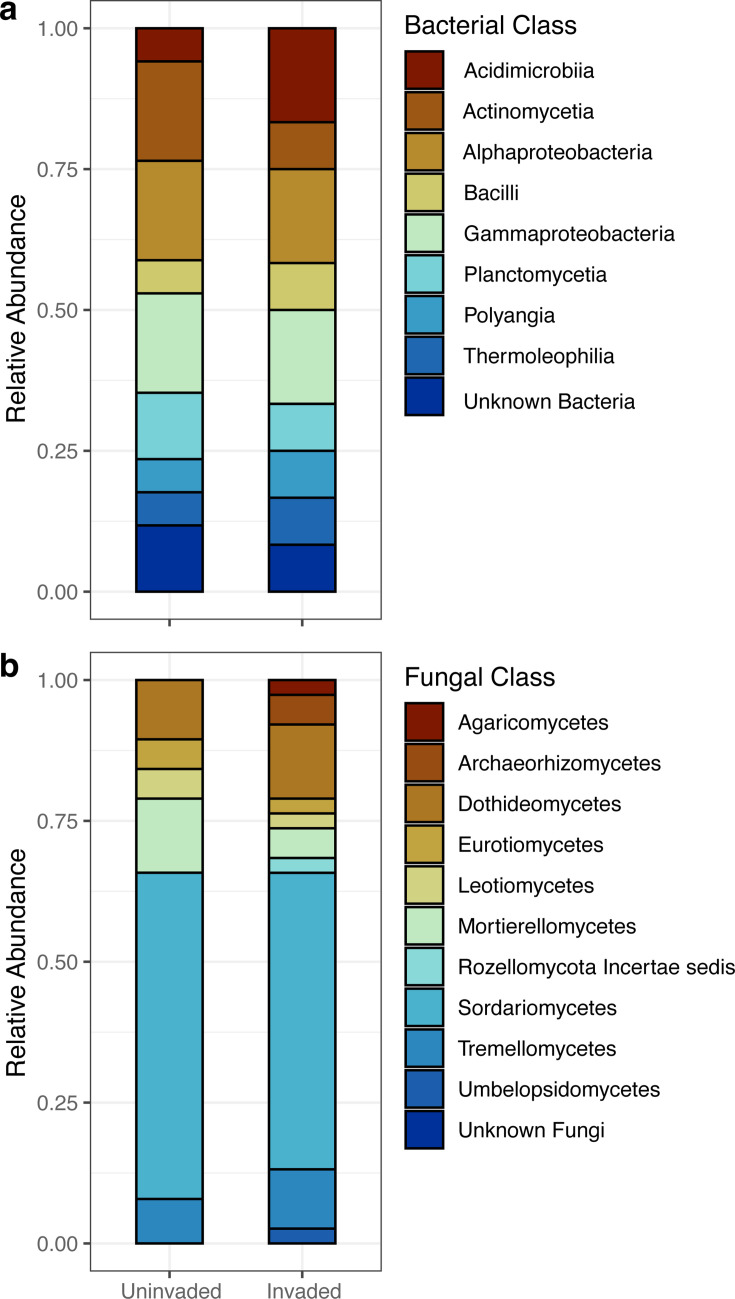
Relative abundance of core sequences predicted to be under positive environmental selection. Relative abundance of bacterial (**a**) and fungal (**b**) sequence variants grouped at the level of taxonomic class is displayed for bulk uninvaded and bulk invaded soils (bacterial *n* = 116 uninvaded soils and 120 invaded soils; fungal *n* = 119 uninvaded and invaded soil). Sequences were filtered to represent only members of a putative core microbiome and predicted to be under positive selection based on their status as over-occupant relative to their mean abundance. Shading denotes taxonomic class.

Core microbial communities were defined for invaded, uninvaded, and rhizosphere soils to identify taxa consistently or uniquely selected for by wavyleaf basketgrass invasion. Occupancy-abundance curves and a neutral model of community assembly were used to identify putatively core sequence variants (Fig. 7; Table 2). A subset of these core sequence variants exhibiting higher sample occupancy than expected by chance based on their mean relative abundance was considered to represent microbes preferentially selected for by the soil and/or plant environments. The unique and shared status of core sequences from invaded and uninvaded bulk soils was visualized in a Euler diagram (Fig. 7g and h), and the relative abundance of their aggregate core and overoccupant taxonomic classes varied slightly in representation between bulk soil types (Fig. 8). Various core microbiome definitions have been used to identify putatively important plant-microbe relationships, including for invasive seagrass and buffelgrass (69, 70), and are most commonly based on cutoff points for sample occupancy or sequence relative abundance. In the present study, we used a core definition based on sequence variant contributions to community dissimilarity, allowing us to define a fraction of the soil microbiota that could be relevant to biotic homogenization processes. By further narrowing this list to include only over-occupant sequences, we present a list of taxa which appeared responsive to the deterministic processes or selective filters applied by wavyleaf basketgrass invasion. The predicted taxonomy of sequences identified in these results (Table S2), as well as those sequences appearing to be amplified in rhizospheres based on ANCOM-BC2 results, should help focus additional research on the function of microbes that are most closely associated with wavyleaf basketgrass invasion. Selective isolation and characterization of these fungal and bacterial taxa could speed efforts to understand the ecological role of environmental microbes amplified during wavyleaf basketgrass spread.

**TABLE 2:** Neutral model estimates from observed sequence variant occupancy-abundance curves

Parameter	Bacterial communities in soil condition			Fungal communities in soil condition		
	Uninvaded (%)	Invaded (%)	Rhizosphere (%)	Uninvaded (%)	Invaded (%)	Rhizosphere (%)
Migration rate parameter	0.043	0.050	0.042	0.006	0.007	0.012
*R* ^2^	0.869	0.882	0.847	0.227	0.272	0.409
Total number of sequence variants	11,468	11,507	8,774	7,070	7,451	8,360
Variants above prediction	363 (3.17)	413 (3.59)	141 (1.61)	377 (5.33)	435 (5.84)	420 (5.02)
Variants below prediction	202 (1.76)	245 (2.13)	142 (1.62)	168 (2.38)	152 (2.04)	165 (1.97)
Variants assigned to core community	61 (0.53)	44 (0.38)	43 (0.49)	52 (0.74)	51 (0.68)	61 (0.73)

### Conclusion

This study showed that wavyleaf basketgrass invasion into Mid-Atlantic forests was associated with changes in the diversity and composition of soil microbiomes. Most notably, soil microbial communities experiencing invasion were homogenized over hundreds of kilometers and across sites in both deciduous and pine forest ecosystems. Wavyleaf basketgrass appeared to recruit and amplify specific soil microbes, and these microbial associates may facilitate continued invasion success or lead to additional invasion impacts on the forest community, perhaps by altering nutrient cycling processes or native plant-microbe interaction networks. Further work is needed to identify the function of individual microbes associated with wavyleaf basketgrass invasion, the mechanisms by which wavyleaf basketgrass influences microbial communities, and whether similar invasion effects are observed in more recently colonized sites. These questions increase in importance as the invasive wavyleaf basketgrass range continues to expand throughout the eastern United States.

## MATERIALS AND METHODS

### Site selection and soil collection

Sampling locations with extensive wavyleaf basketgrass invasion documented for at least 5 years were chosen for this study. From May to August 2019, bulk soils were collected from four locations in Maryland (Gunpowder Falls State Park, Patapsco Valley State Park, Liberty Reservoir, and Middle Patuxent Environmental Area) and two locations in Virginia (Fraser Preserve and Shenandoah National Park). From June to July 2022, bulk soils and rhizosphere samples were collected from five locations in Virginia (Piney Grove Reservoir, Beldor Hollow, Smithsonian Conservation Biology Institute, Berry Farm Trail, and Williams Woods) and one location in Maryland (Woodstock Area of Patapsco Valley State Park) (Fig. 1; Table 3).

**TABLE 3:** Locations of *Oplismenus undulatifolius* sites used in this study

Year	Location	County	State	Latitude	Longitude
2019	Gunpowder Falls State Park	Baltimore	MD	39.59473402	−76.67260003
2019	Patapsco Valley State Park	Howard	MD	39.33275403	−76.87252896
2019	Liberty Reservoir	Carroll	MD	39.404029	−76.864483
2019	Middle Patuxent Environmental Area	Howard	MD	39.216656	−76.902087
2019	Fraser Preserve	Fairfax	VA	39.047749	−77.308081
2019	Shenandoah National Park	Rockingham	VA	38.3634667	−78.5763333
2022	Woodstock area of Patapsco Valley State Park	Howard	MD	39.333222	−76.782965
2022	William S. D. Woods Natural Heritage Area	Albemarle	VA	37.974339	−78.593264
2022	Berry Farm Trail	Augusta	VA	38.207552	−79.041658
2022	Beldor Hollow	Greene	VA	38.315604	−78.630418
2022	Piney Grove Preserve	Sussex	VA	36.99907	−77.06974
2022	Smithsonian Conservation Biology Institute	Warren	VA	38.870702	−78.161315

In 2019, we sampled from 10 wavyleaf basketgrass-dominated (hereafter, “invaded”) plots (4 m^2^) and 10 plots with little to no wavyleaf basketgrass cover (“uninvaded”) at each location, all of which occurred in deciduous hardwood forests dominated by an overstory of *Liriodendron tulipifera*, *Carpinus caroliniana*, *Lindera benzoin*, and *Fagus grandifolia*. Invaded locations had greater than 55% *O. undulatifolius* cover, except at Shenandoah, where *O. undulatifolius* cover was greater than 45%, whereas uninvaded plots had less than 5% *O. undulatifolius* cover. At all invaded plots, *O. undulatifolius* was the most abundant plant species present. After locating the first plot, the others were chosen by selecting a random direction, walking 15 m from the current plot, and continuing until reaching a suitable invaded or uninvaded patch. All plots were at least 15 m from trails and at least 15 m from each other, except at Middle Patuxent Environmental Area, where the minimum distance between plots of 10 m was due to the limited distribution of wavyleaf basketgrass in the area and the location’s small size. Samples were collected from the top 10 cm of soil at the center and four corners of each plot using a trowel. Soil from all five subsamples per plot was mixed and placed into sterile plastic bags. Sampling equipment was sterilized between plots with a dilute suspension of sodium hypochlorite and 0.1% Tween-80 detergent. The samples were placed on ice until transport to the lab, where they were stored at 4**°**C during processing.

In 2022, we identified 10 sites within each of the six locations with >80% wavyleaf basketgrass groundcover (invaded) and paired each with a site located at least 10 m away with no wavyleaf basketgrass plants identified (uninvaded). At each of these 20 sites, we combined five soil cores (10 cm depth, 3.1 cm diameter) collected at each corner and in the center of a 4 m^2^ quadrat into a single bulk soil sample. All collecting materials were cleaned with 70% ethanol between each quadrat, and leaf litter was removed prior to collecting each soil core. We also collected soil from the wavyleaf basketgrass rhizosphere within each of the quadrats established for invaded soil sampling. At each quadrat in an invaded site, rhizosphere soils were pooled from the root systems of four to five wavyleaf basketgrass plants growing within the 4 m^2^ area. Roots were carefully dislodged from the soil and neighboring roots, gently shaken to remove bulk soil, and placed in a sterilized paper bag within a sterile plastic bag. All soil samples were transported to the lab in a cooler with ice packs, where the material was stored at 4°C during processing. Rhizosphere samples were collected from the soil that remained adhered to the roots (71–73).

### Plant community sampling

Plant species richness and ground cover were recorded for understory vegetation (<2 m tall) in each 4 m^2^ plot at locations sampled in 2019. The cover of each species was estimated to the nearest 10%, and total plot cover was calculated as the sum of all species covers. Plants that could not be identified in the field were collected from outside the plot for later identification (74).

### Soil chemical analysis

Soil chemical analyses were conducted on a random subsample of soils including five invaded and five uninvaded plots at each field location visited in 2019. Samples were air-dried, ground, and sieved to 2 mm (Brookside Laboratories Inc, 2021). Soils were analyzed for total exchange capacity (75), pH (76, 77), and the carbon to nitrogen ratio (78). Additionally, pH was recorded for all soils collected in 2021.

### DNA extraction, sequencing, and analysis

Homogenized soil samples were passed through a 2 mm sieve to remove roots and rocks before storage at −80°C. Prior to DNA extraction, soils were air-dried under sterile cheesecloth on a laboratory bench for 24 h at room temperature. Total genomic DNA was extracted from ~80 mg of air-dried soil using the PowerSoil Pro Kit (Qiagen, CA, USA) according to the manufacturer’s protocol with the addition of a final isopropanol precipitation step. DNA concentrations and quality were checked using a Nanodrop 2000 spectrophotometer (ThermoFisher, Waltham, MA). Prokaryotic and fungal communities were characterized by amplicon sequencing. Library preparation and Illumina sequencing were performed at the University of Minnesota Genomics Center. Amplicons of the fungal internal transcribed spacer region of ribosomal DNA (ITS2, primers: 5.8SR TCGATGAAGAACGCAGCG and ITS4 TCCTCCGCTTATTGATATGC) and of the bacterial 16S ribosomal DNA (16S V4, primers: 515F GTGCCAGCMGCCGCGGTAA and 806R GGACTACHVGGGTWTCTAAT) were pooled and sequenced with an Illumina MiSeq (CA, USA) generating 300-bp paired-end reads (24,969,090 total read pairs) (79–81).

Average read quality was calculated for each sample with FastQC v.0.12 (R1 quality ≥ 26, R2 quality ≥ 21) (82), and based on predicted adapter content, 5′ read ends were trimmed by 20 bp for 16S amplicons and 22 bp for ITS2 amplicons using Cutadapt v.4.0 (83). Sequences were imported into QIIME2, and read quality was assessed visually with interactive quality plots using a random subsample of 10,000 sequences, so that low-quality regions of sequence could be excluded during later processing steps (84). Read lengths were subsequently truncated during denoising and merging with dada2 in order to remove low-quality base pairs from 16S (Read 1: 260 bp retained, Read 2: 190 bp retained) and ITS2 amplicons (Read 1: 200 bp retained, Read 2: 180 bp retained) (85). Exact sequence variants resulting from these processing steps were used for all downstream analyses. Taxonomic assignments were made using naïve Bayes classifiers trained with the Greengenes2 and UNITE v9 (released 29 November 2022) databases for 16S and ITS2 sequences, respectively (86, 87). For phylogenetic analysis, sequences were aligned using mafft (88), and midpoint-rooted trees were generated with fasttree (89). Rarefaction curves were generated to observe sequence richness estimates (90). All samples were rarified to a depth of 4,000 sequences prior to statistical analysis. This threshold was selected based on the distribution of sequence reads per sample and an examination of rarefaction curves (Fig. S1). Point estimates at this depth allowed for the retention of most individual samples while still accurately representing the relationship of asymptotic richness estimates. Five 16S and four ITS2 samples were omitted from analysis for having fewer than 4,000 sequence reads.

Alpha diversity (Faith’s phylogenetic diversity, Shannon’s H′, Pielou’s E, and observed sequence counts) and beta diversity (phylogenetically weighted UniFrac) were calculated using the diversity module in QIIME2. These metrics and their underlying feature tables were exported for analysis in R v.4.4.3 (91). Linear modeling, mean estimation, and pairwise contrasts were performed using the “lme4,” “lmerTest,” and “emmeans” R packages (92, 93). Alpha diversity metrics were analyzed with linear mixed models using a restricted maximum likelihood method, as implemented in the lmer function of “lme4.” Soil condition was included as a fixed effect, and a random effect was incorporated to account for different sampling locations. Each year of data was modeled independently due to the unbalanced sampling of rhizosphere soils. The significance of soil condition as a predictor was determined for each model using analysis of variance performed using Wald *X*^2^ tests with Satterthwaite approximated degrees of freedom. Random effect significance for sampling location was assessed using likelihood-ratio tests implemented in the ranova function from “lmerTest” (94). Soil condition means, standard errors, and 95% confidence intervals were calculated in order to perform pairwise comparisons between uninvaded, invaded, and rhizosphere samples, with direct comparisons drawn only between samples collected in the same year. Soil chemistry and plant community data from invaded and uninvaded plots were contrasted with non-parametric Kruskal-Wallis tests and examined for Spearman correlation with phylogenetic alpha diversity. Beta diversity recorded as weighted UniFrac distances (95) was calculated between all pairs of samples and analyzed with permutational analysis of variance, permutational analysis of dispersion, and non-metric multidimensional scaling plots created with the R package “vegan” (96). Permutations were performed using sample location as a blocking factor, so that estimates of variation attributed to each soil condition were effectively nested within both field site and year of sampling. NMDS was performed using *k* = 3 dimensions.

Differences in the relative abundance of specific sequences among soil conditions observed in 2022 were determined using ANCOM-BC2, which is based on a generalized linear modeling framework (97). Sampling site and soil condition were included as fixed effects and pairwise comparisons between soil conditions were calculated to provide *P*-values adjusted according to Holm (98). This analysis incorporated default ANCOM-BC2 procedures for a sensitivity analysis that limits false positives by testing for the effect of pseudo-count additions on low abundance sequences. Results reported include only those significant differences that were below the Holm adjusted significance level and invariant with changes in pseudo-count magnitude.

Core microbiomes were defined for each soil condition in order to identify taxa putatively important to wavyleaf basketgrass invasion processes for future study. The approach used was based on the Sloan neutral model of community assembly applied to occupancy-abundance relationships and implemented in R by Burns (99). In brief, soil sample occupancy was determined for each sequence variant (proportion of samples where a sequence was present), an overall migration rate parameter (*m*) was estimated from sequence relative abundances using non-linear least squares regression, and expected occupancy predictions were made for each sequence using a maximum likelihood model based on estimated migration parameter and mean sequence relative abundances within occupied samples. A threshold for inclusion in the core microbiomes was established using individual sequence contributions to Bray-Curtis community dissimilarity, following the procedures of Shade and Stopnisek (100) to identify sequences whose inclusion in Bray-Curtis calculations contributed at least a 5% increase in the dissimilarities observed, effectively identifying a fraction of the community with both high occupancy and mean relative abundance that could be related to biotic homogenization. Sequences were further prioritized from this core fraction if they were also observed to be over-occupant, in other words, having an observed sample occupancy rate greater than the upper prediction limit of a 95% confidence interval based on the fitted Sloan neutral model. These sequences and the taxa they represent were considered putatively under environmental and/or host selection (99) and therefore relevant to any deterministic community assembly processes influenced by wavyleaf basketgrass invasion. The subset of core, over-occupant sequences from invaded and uninvaded soils was compared to identify shared and unique sequence variants and then aggregated at the taxonomic level of class to examine higher-level changes in relative abundance. All figures were generated using R packages “ggplot2,” “ggpubr,” and “scico” (101–103).

## Data Availability

Amplicon sequencing data have been deposited in GenBank Sequence Read Archive under BioProject ID PRJNA1211650.

## SUPPLEMENTAL MATERIAL

Supplemental tables and figure msphere.00895-25-s0001.docx ANOVA outputs, putative core taxa, and rarefaction curves.

ASM does not own the copyrights to Supplemental Material that may be linked to, or accessed through, an article. The authors have granted ASM a non-exclusive, world-wide license to publish the Supplemental Material files. Please contact the corresponding author directly for reuse.
