# Supplementary material for: Wavyleaf basketgrass (Oplismenus undulatifolius) invasion is associated with changes in soil microbial communities
Source: mSphere. 2026 Apr 29;11(5):e00895-25. doi: 10.1128/msphere.00895-25 (PMC13203986; doi:10.1128/msphere.00895-25)

**Supplemental Materials:**

**Wavyleaf basketgrass (*Oplismenus undulatifolius*) invasion is associated with changes in soil microbial communities**

Michael R. Fulcher^a#^, Anthony Tritz^b^, Vanessa Beauchamp^b^, Carrie A. Wu^c^

a Foreign Disease-Weed Science Research Unit, Agricultural Research Service, U.S. Department of Agriculture, Frederick, Maryland, USA

b Department of Biological Sciences, Towson University, Maryland, USA

c Department of Biology, University of Richmond, Virginia, USA

# Address correspondence to Michael R. Fulcher, [michael.fulcher@usda.gov](mailto:michael.fulcher@usda.gov)

**Supplemental Table 1. Analysis of variance results for mixed linear model analysis of microbial alpha diversity measurements using soil condition as a predictor.**

|  | Bacteria | | | | | |  | Fungi | | | | | |
| --- | --- | --- | --- | --- | --- | --- | --- | --- | --- | --- | --- | --- | --- |
|  | 2019 | | | 2022 | | |  | 2019 | | | 2022 | | |
|  | χ^2^ / LRT^z^ | D.f. | *p* | χ^2^ | D.f. | *p* |  | χ^2^ | D.f. | *p* | χ^2^ | D.f. | *p* |
| **Faith's PD** |  |  |  |  |  |  |  |  |  |  |  |  |  |
| Condition (fixed) | 0.255 | 1 | 0.613 | 6.943 | 2 | **0.030** |  | 1.737 | 1 | 0.188 | 53.226 | 2 | **<0.001** |
| Location (random) | 0.742 | 1 | 0.389 | 1.750 | 1 | 0.186 |  | 0.428 | 1 | 0.513 | 4.106 | 1 | **0.043** |
| **Shannon's H`** |  |  |  |  |  |  |  |  |  |  |  |  |  |
| Condition (fixed) | 0.232 | 1 | 0.630 | 4.968 | 2 | 0.083 |  | 4.060 | 1 | **0.040** | 38.097 | 2 | **<0.001** |
| Location (random) | <0.001 | 1 | 1.000 | 0.337 | 1 | 0.562 |  | <0.001 | 1 | 1.000 | 4.981 | 1 | **0.026** |
| **Pielou's E** |  |  |  |  |  |  |  |  |  |  |  |  |  |
| Condition (fixed) | 0.980 | 1 | 0.322 | 3.735 | 2 | 0.155 |  | 7.763 | 1 | **0.005** | 12.840 | 2 | **0.002** |
| Location (random) | 3.584 | 1 | 0.058 | 30.974 | 1 | **<0.001** |  | <0.001 | 1 | 1.000 | 1.479 | 1 | 0.224 |
| **Observed Sequence Variants** |  |  |  |  |  |  |  |  |  |  |  |  |  |
| Condition (fixed) | 0.013 | 1 | 0.909 | 5.594 | 2 | 0.061 |  | 0.052 | 1 | 0.820 | 39.650 | 2 | **<0.001** |
| Location (random) | <0.001 | 1 | 1.000 | 0.290 | 1 | 0.590 |  | 0.376 | 1 | 0.540 | 3.042 | 1 | 0.081 |

^z^ Wald Chi-square test statistic for standard ANOVA, and likelihood-ratio test for random effect analysis

**Supplemental Table 2. Taxonomic assignment of putative core microbes occurring at higher than predicted rates in soil microbial communities.**

Core membership was predicted for exact sequence variants found in each soil type. Taxonomic assignments and confidence in the lowest level taxonomy prediction are presented based on the results of naïve Bayesian classifiers trained on GreenGenes2 (16S) and UNITE (ITS2).

| Core Membership | Exact Sequence Variant ID | Kingdom | Phylum | Class | Order | Family | Genus | Species | Confidence |
| --- | --- | --- | --- | --- | --- | --- | --- | --- | --- |
| Uninvaded | e8820a8b0ccb14b286019c21e8bfcf00 | Bacteria | Firmicutes D | Bacilli | Bacillales B 310392 | Bacillaceae G 310392 | Bacillus A | NA | 1.00 |
| Uninvaded | 5d2d2c381e9dc99e5ac57ed268c8ceee | Bacteria | Planctomycetota | Planctomycetia | Isosphaerales | Isosphaeraceae | NA | NA | 1.00 |
| Uninvaded | 3592495878636d0db098b56d90c49f70 | Bacteria | Actinobacteriota | Actinomycetia | Mycobacteriales | Jatrophihabitantaceae | Jatrophihabitans 372606 | NA | 0.95 |
| Uninvaded | 4081533e524226f7ed5eecdadb7a2c91 | Bacteria | Actinobacteriota | Thermoleophilia | Gaiellales | Gaiellaceae | Gaiella | NA | 0.90 |
| Uninvaded | 14f161a1af462a03d7f19432cfdb68c6 | Bacteria | Proteobacteria | Alphaproteobacteria | Rhizobiales A 504705 | Xanthobacteraceae 503485 | Bradyrhizobium | NA | 0.99 |
| Uninvaded | ac7c8e9de2cfd6f893720cc7118ad387 | Bacteria | Proteobacteria | Gammaproteobacteria | Burkholderiales 592522 | Burkholderiaceae A 592522 | NA | NA | 1.00 |
| Uninvaded | 9e19be3efeb471bc05e6818a949eb529 | Bacteria | Actinobacteriota | Actinomycetia | Actinomycetales | Cellulomonadaceae | Cellulomonas | NA | 0.99 |
| Uninvaded | 906b2567ddda9890572015419c397fd6 | Bacteria | Proteobacteria | Gammaproteobacteria | Burkholderiales 597441 | SHXO01 | SHXO01 | NA | 0.98 |
| Uninvaded | 84b0a2a8482e5f8986477120b857aa77 | Bacteria | Actinobacteriota | NA | NA | NA | NA | NA | 0.99 |
| Uninvaded | bce5d153cbb40518fcd3c58a399d40b4 | Bacteria | Proteobacteria | Alphaproteobacteria | NA | NA | NA | NA | 1.00 |
| Uninvaded | 42003fb3810fd360c92cdbc0b18b246c | Bacteria | Planctomycetota | Planctomycetia | Isosphaerales | Isosphaeraceae | Aquisphaera | NA | 0.88 |
| Uninvaded | 41f8a5aac40e3e83cc5955dc4f0f7377 | Bacteria | Actinobacteriota | Actinomycetia | Propionibacteriales | Nocardioidaceae | Nocardioides A 392796 | Nocardioides A 392796 cynanchi | 0.79 |
| Uninvaded | 5bd651d04555a5bcc20ba4fc9c8676f3 | Bacteria | Myxococcota A 473307 | Polyangia 463783 | Polyangiales | Polyangiaceae | NA | NA | 0.96 |
| Uninvaded | ba4d475c9d5296670d3625f11aa91404 | Bacteria | Actinobacteriota | Acidimicrobiia 401430 | Acidimicrobiales | Ilumatobacteraceae | NA | NA | 0.74 |
| Uninvaded | c43a0af0fa9386431921e6a7128e62c0 | Bacteria | Proteobacteria | Alphaproteobacteria | NA | NA | NA | NA | 1.00 |
| Uninvaded | 06c954688f1e95e67118fb7df5eb1ac1 | Bacteria | Proteobacteria | Gammaproteobacteria | Burkholderiales 597441 | SHXO01 | SHXO01 | NA | 0.73 |
| Uninvaded | 079240a1ba113328cf31bd0c39964cfb | Fungi | Mortierellomycota | Mortierellomycetes | Mortierellales | Mortierellaceae | Podila | Podila minutissima | 0.86 |
| Uninvaded | fc4a5d7e90512d7424fb2e939f0d79dd | Fungi | Basidiomycota | Tremellomycetes | Filobasidiales | Piskurozymaceae | Solicoccozyma | Solicoccozyma terricola | 1.00 |
| Uninvaded | 7fbb93d82e4abea98be2b43aa3e311fd | Fungi | Ascomycota | Dothideomycetes | Capnodiales | Cladosporiaceae | Cladosporium | NA | 1.00 |
| Uninvaded | 6546a19107339a6e4e7c757772ea128f | Fungi | Mortierellomycota | Mortierellomycetes | Mortierellales | Mortierellaceae | NA | NA | 1.00 |
| Uninvaded | c63041a6bbb95095ee08dcf911c22fdc | Fungi | Ascomycota | Dothideomycetes | Pleosporales | Phaeosphaeriaceae | Paraphoma | Paraphoma chrysanthemicola | 0.97 |
| Uninvaded | a702dbddbbc64f733218e0bb7a308931 | Fungi | Ascomycota | Dothideomycetes | Pleosporales | Didymellaceae | NA | NA | 1.00 |
| Uninvaded | 53b7b24ed7ecf1fd89d6e62031cbb420 | Fungi | Ascomycota | Sordariomycetes | Sordariales | Chaetomiaceae | Humicola | Humicola fuscoatra | 0.85 |
| Uninvaded | 34d5a0367b1fc5f12f0c456c51accc01 | Fungi | Ascomycota | Sordariomycetes | Hypocreales | Clavicipitaceae | Keithomyces | Keithomyces carneus | 0.90 |
| Uninvaded | df9e2763afcf9404f2ad3953676fb6e2 | Fungi | Mortierellomycota | Mortierellomycetes | Mortierellales | Mortierellaceae | Mortierella | NA | 0.81 |
| Uninvaded | 33adac43d1106225f6f44a857f3b681b | Fungi | Ascomycota | Sordariomycetes | Hypocreales | Nectriaceae | Ilyonectria | Ilyonectria mors-panacis | 0.97 |
| Uninvaded | ba14b83d3509898c8d33ee1642f84354 | Fungi | Ascomycota | Sordariomycetes | Hypocreales | Hypocreaceae | Trichoderma | NA | 1.00 |
| Uninvaded | 7371fb25c49ccb78a47f5891aa85a994 | Fungi | Ascomycota | Sordariomycetes | Sordariales | Sordariales fam Incertae sedis | Staphylotrichum | Staphylotrichum coccosporum | 1.00 |
| Uninvaded | 901fa3de4a674df8e0566718f787de2c | Fungi | Ascomycota | Sordariomycetes | Hypocreales | Clavicipitaceae | Keithomyces | NA | 0.82 |
| Uninvaded | c91b38d350962b22a24ad4beb5481d87 | Fungi | Ascomycota | Sordariomycetes | Xylariales | Microdochiaceae | Idriella | Idriella lunata | 0.84 |
| Uninvaded | 44b248d57966ef3159c6d80c7e9d9424 | Fungi | Ascomycota | Sordariomycetes | Hypocreales | Bionectriaceae | Lasionectriopsis | Lasionectriopsis spinosa | 1.00 |
| Uninvaded | 4360403cd3e59f847933844e89565fb8 | Fungi | Ascomycota | Sordariomycetes | Hypocreales | Bionectriaceae | Clonostachys | NA | 0.98 |
| Uninvaded | a22a291e190fa60f055bbc68659fb455 | Fungi | Ascomycota | Sordariomycetes | Sordariales | Chaetomiaceae | Humicola | Humicola fuscoatra | 0.88 |
| Uninvaded | 11d3077e6c143703f11d4bd218292d36 | Fungi | Ascomycota | Sordariomycetes | Sordariales | NA | NA | NA | 0.98 |
| Uninvaded | 0467b75a51841d838b050df0f247c0bf | Fungi | Mortierellomycota | Mortierellomycetes | Mortierellales | Mortierellaceae | Podila | Podila horticola | 1.00 |
| Uninvaded | 4740ac8556d9433dff14c37d15ca1fda | Fungi | Ascomycota | Sordariomycetes | Chaetosphaeriales | Chaetosphaeriaceae | Chloridium | Chloridium aseptatum | 1.00 |
| Uninvaded | d516428906b18863774f6dd7b11a1036 | Fungi | Ascomycota | Eurotiomycetes | Eurotiales | Aspergillaceae | NA | NA | 1.00 |
| Uninvaded | 24e74d80a14c45a5cf5d90ec5a781cc7 | Fungi | Ascomycota | Sordariomycetes | Hypocreales | Nectriaceae | Fusarium | Fusarium solani | 0.75 |
| Uninvaded | dd3ed6d9d221606f163622587d92c714 | Fungi | Ascomycota | Leotiomycetes | Thelebolales | Pseudeurotiaceae | Geomyces | Geomyces sp | 0.95 |
| Uninvaded | 2ada98f50bd767e818ac1b1f2449170b | Fungi | Ascomycota | Eurotiomycetes | Eurotiales | Aspergillaceae | Penicillium | Penicillium ochotense | 0.88 |
| Uninvaded | d1eab991efac55837e9a6b2f9f0d997b | Fungi | Ascomycota | Sordariomycetes | Hypocreales | Clavicipitaceae | Metapochonia | Metapochonia rubescens | 0.97 |
| Uninvaded | 60ee4f97b4ac5a0cd3c1d6f24b02547f | Fungi | Basidiomycota | Tremellomycetes | Tremellales | Tremellaceae | Tremella | Tremella sp | 1.00 |
| Uninvaded | f9e6c2e90cd54eed887bda741e41d270 | Fungi | Ascomycota | Sordariomycetes | Hypocreales | Hypocreaceae | Trichoderma | Trichoderma pubescens | 1.00 |
| Uninvaded | 64d989fd28f1ef848f4e6f19b9101282 | Fungi | Basidiomycota | Tremellomycetes | Trichosporonales | Trichosporonaceae | Apiotrichum | Apiotrichum dehoogii | 0.76 |
| Uninvaded | 2db3a4c9d2ad86389ee9d49baff7662a | Fungi | Ascomycota | Sordariomycetes | Hypocreales | Nectriaceae | Dactylonectria | Dactylonectria anthuriicola | 1.00 |
| Uninvaded | 9be4cea723dd381390e1ba4d3b3ca39a | Fungi | Ascomycota | Sordariomycetes | Sordariales | Chaetomiaceae | Humicola | Humicola malaysiensis | 0.98 |
| Uninvaded | 9ec9c6007520a953344c67b0f1a9eabb | Fungi | Ascomycota | Sordariomycetes | Hypocreales | Clavicipitaceae | NA | NA | 1.00 |
| Uninvaded | a0c88a5edb708f0ff3cbd8019d758296 | Fungi | Ascomycota | Leotiomycetes | Thelebolales | Pseudeurotiaceae | Pseudogymnoascus | Pseudogymnoascus roseus | 0.87 |
| Uninvaded | 9d90edd8736d2f2a40a88ff23e08aa1c | Fungi | Ascomycota | Sordariomycetes | Sordariales | Sordariales fam Incertae sedis | Cordana | Cordana ellipsoidea | 0.99 |
| Uninvaded | ac35b0cb8561b769c55e16435f8aafe0 | Fungi | Ascomycota | Dothideomycetes | Pleosporales | Melanommataceae | Pleotrichocladium | Pleotrichocladium opacum | 0.94 |
| Uninvaded | 29da4f394c5c9a7396da048d72ee298f | Fungi | Ascomycota | Sordariomycetes | Hypocreales | Clavicipitaceae | Metapochonia | NA | 0.76 |
| Uninvaded | f3919e1ed71fb491b939bf8052050618 | Fungi | Ascomycota | Sordariomycetes | Glomerellales | Glomerellales fam Incertae sedis | Glomerellales gen Incertae sedis | Glomerellales sp | 0.88 |
| Uninvaded | 00f7b2d74cdf87e4578d44c7ac7afc4e | Fungi | Ascomycota | Sordariomycetes | Hypocreales | Nectriaceae | Mariannaea | Mariannaea camptospora | 1.00 |
| Uninvaded | c706d5ba296460e895e1053fbdd103f6 | Fungi | Mortierellomycota | Mortierellomycetes | Mortierellales | Mortierellaceae | Gryganskiella | Gryganskiella cystojenkinii | 1.00 |
| Invaded | e8820a8b0ccb14b286019c21e8bfcf00 | Bacteria | Firmicutes D | Bacilli | Bacillales B 310392 | Bacillaceae G 310392 | Bacillus A | NA | 1.00 |
| Invaded | 5d2d2c381e9dc99e5ac57ed268c8ceee | Bacteria | Planctomycetota | Planctomycetia | Isosphaerales | Isosphaeraceae | NA | NA | 1.00 |
| Invaded | 3592495878636d0db098b56d90c49f70 | Bacteria | Actinobacteriota | Actinomycetia | Mycobacteriales | Jatrophihabitantaceae | Jatrophihabitans 372606 | NA | 0.95 |
| Invaded | 4081533e524226f7ed5eecdadb7a2c91 | Bacteria | Actinobacteriota | Thermoleophilia | Gaiellales | Gaiellaceae | Gaiella | NA | 0.90 |
| Invaded | 14f161a1af462a03d7f19432cfdb68c6 | Bacteria | Proteobacteria | Alphaproteobacteria | Rhizobiales A 504705 | Xanthobacteraceae 503485 | Bradyrhizobium | NA | 0.99 |
| Invaded | ac7c8e9de2cfd6f893720cc7118ad387 | Bacteria | Proteobacteria | Gammaproteobacteria | Burkholderiales 592522 | Burkholderiaceae A 592522 | NA | NA | 1.00 |
| Invaded | 9e19be3efeb471bc05e6818a949eb529 | Bacteria | Actinobacteriota | Actinomycetia | Actinomycetales | Cellulomonadaceae | Cellulomonas | NA | 0.99 |
| Invaded | 906b2567ddda9890572015419c397fd6 | Bacteria | Proteobacteria | Gammaproteobacteria | Burkholderiales 597441 | SHXO01 | SHXO01 | NA | 0.98 |
| Invaded | 84b0a2a8482e5f8986477120b857aa77 | Bacteria | Actinobacteriota | NA | NA | NA | NA | NA | 0.99 |
| Invaded | 30f1fe30bb6fa44028182b7ecba785ce | Bacteria | Actinobacteriota | Acidimicrobiia 401430 | Acidimicrobiales | Ilumatobacteraceae | NA | NA | 0.76 |
| Invaded | 42003fb3810fd360c92cdbc0b18b246c | Bacteria | Planctomycetota | Planctomycetia | Isosphaerales | Isosphaeraceae | Aquisphaera | NA | 0.88 |
| Invaded | 5bd651d04555a5bcc20ba4fc9c8676f3 | Bacteria | Myxococcota A 473307 | Polyangia 463783 | Polyangiales | Polyangiaceae | NA | NA | 0.96 |
| Invaded | ba4d475c9d5296670d3625f11aa91404 | Bacteria | Actinobacteriota | Acidimicrobiia 401430 | Acidimicrobiales | Ilumatobacteraceae | NA | NA | 0.74 |
| Invaded | 06c954688f1e95e67118fb7df5eb1ac1 | Bacteria | Proteobacteria | Gammaproteobacteria | Burkholderiales 597441 | SHXO01 | SHXO01 | NA | 0.73 |
| Invaded | 231afad83a5acae555314a9654703d9a | Bacteria | NA | NA | NA | NA | NA | NA | 1.00 |
| Invaded | 079240a1ba113328cf31bd0c39964cfb | Fungi | Mortierellomycota | Mortierellomycetes | Mortierellales | Mortierellaceae | Podila | Podila minutissima | 0.86 |
| Invaded | fc4a5d7e90512d7424fb2e939f0d79dd | Fungi | Basidiomycota | Tremellomycetes | Filobasidiales | Piskurozymaceae | Solicoccozyma | Solicoccozyma terricola | 1.00 |
| Invaded | 7fbb93d82e4abea98be2b43aa3e311fd | Fungi | Ascomycota | Dothideomycetes | Capnodiales | Cladosporiaceae | Cladosporium | NA | 1.00 |
| Invaded | 6546a19107339a6e4e7c757772ea128f | Fungi | Mortierellomycota | Mortierellomycetes | Mortierellales | Mortierellaceae | NA | NA | 1.00 |
| Invaded | b380fc8440a0bfececf28e62485bd49a | Fungi | Ascomycota | Dothideomycetes | Pleosporales | Didymosphaeriaceae | Paraconiothyrium | NA | 0.96 |
| Invaded | a702dbddbbc64f733218e0bb7a308931 | Fungi | Ascomycota | Dothideomycetes | Pleosporales | Didymellaceae | NA | NA | 1.00 |
| Invaded | 53b7b24ed7ecf1fd89d6e62031cbb420 | Fungi | Ascomycota | Sordariomycetes | Sordariales | Chaetomiaceae | Humicola | Humicola fuscoatra | 0.85 |
| Invaded | d1ec54f0d0f34aea74ee251793781c2d | Fungi | Mucoromycota | Umbelopsidomycetes | Umbelopsidales | Umbelopsidaceae | Umbelopsis | Umbelopsis dimorpha | 1.00 |
| Invaded | f3a90845827cb8809d121fafe18c20ee | Fungi | Ascomycota | Sordariomycetes | Sordariales | Chaetomiaceae | Humicola | Humicola fuscoatra | 0.94 |
| Invaded | 34d5a0367b1fc5f12f0c456c51accc01 | Fungi | Ascomycota | Sordariomycetes | Hypocreales | Clavicipitaceae | Keithomyces | Keithomyces carneus | 0.90 |
| Invaded | 33adac43d1106225f6f44a857f3b681b | Fungi | Ascomycota | Sordariomycetes | Hypocreales | Nectriaceae | Ilyonectria | Ilyonectria mors-panacis | 0.97 |
| Invaded | 49666b838337ef4553cc513fa364e83f | Fungi | Ascomycota | Dothideomycetes | Pleosporales | Phaeosphaeriaceae | Phaeosphaeria | NA | 1.00 |
| Invaded | a052ce486397ec3beae9580defb78c64 | Fungi | Ascomycota | Sordariomycetes | Hypocreales | Bionectriaceae | Clonostachys | Clonostachys sp | 0.99 |
| Invaded | 7371fb25c49ccb78a47f5891aa85a994 | Fungi | Ascomycota | Sordariomycetes | Sordariales | Sordariales fam Incertae sedis | Staphylotrichum | Staphylotrichum coccosporum | 1.00 |
| Invaded | 901fa3de4a674df8e0566718f787de2c | Fungi | Ascomycota | Sordariomycetes | Hypocreales | Clavicipitaceae | Keithomyces | NA | 0.82 |
| Invaded | 61e163035a5f509a3ccfc8586b990ced | Fungi | Ascomycota | Sordariomycetes | Glomerellales | Plectosphaerellaceae | Longitudinalis | Longitudinalis nabanheensis | 0.77 |
| Invaded | dbb44cac321d71f9c50b99666257d49e | Fungi | Ascomycota | Sordariomycetes | Sordariales | Chaetomiaceae | NA | NA | 0.99 |
| Invaded | c91b38d350962b22a24ad4beb5481d87 | Fungi | Ascomycota | Sordariomycetes | Xylariales | Microdochiaceae | Idriella | Idriella lunata | 0.84 |
| Invaded | 44b248d57966ef3159c6d80c7e9d9424 | Fungi | Ascomycota | Sordariomycetes | Hypocreales | Bionectriaceae | Lasionectriopsis | Lasionectriopsis spinosa | 1.00 |
| Invaded | 4360403cd3e59f847933844e89565fb8 | Fungi | Ascomycota | Sordariomycetes | Hypocreales | Bionectriaceae | Clonostachys | NA | 0.98 |
| Invaded | 4740ac8556d9433dff14c37d15ca1fda | Fungi | Ascomycota | Sordariomycetes | Chaetosphaeriales | Chaetosphaeriaceae | Chloridium | Chloridium aseptatum | 1.00 |
| Invaded | 24e74d80a14c45a5cf5d90ec5a781cc7 | Fungi | Ascomycota | Sordariomycetes | Hypocreales | Nectriaceae | Fusarium | Fusarium solani | 0.75 |
| Invaded | 39cb1e636fe380d4cdafc889cf9e0523 | Fungi | Ascomycota | Eurotiomycetes | Eurotiales | Aspergillaceae | Aspergillus | Aspergillus tardus | 0.99 |
| Invaded | dd3ed6d9d221606f163622587d92c714 | Fungi | Ascomycota | Leotiomycetes | Thelebolales | Pseudeurotiaceae | Geomyces | Geomyces sp | 0.95 |
| Invaded | db43836fd4148e966b71b52cad08f6eb | Fungi | Ascomycota | Sordariomycetes | Xylariales | Microdochiaceae | Idriella | Idriella lunata | 1.00 |
| Invaded | 84f3db5da59b9c684b474da33c6a1178 | Fungi | Ascomycota | Sordariomycetes | Hypocreales | Nectriaceae | Neonectria | NA | 0.78 |
| Invaded | 60ee4f97b4ac5a0cd3c1d6f24b02547f | Fungi | Basidiomycota | Tremellomycetes | Tremellales | Tremellaceae | Tremella | Tremella sp | 1.00 |
| Invaded | 6eb13e9890e6cd15fd3f88653fbfb4a3 | Fungi | Ascomycota | Archaeorhizomycetes | Archaeorhizomycetales | Archaeorhizomycetaceae | Archaeorhizomyces | Archaeorhizomyces sp | 0.95 |
| Invaded | 64d989fd28f1ef848f4e6f19b9101282 | Fungi | Basidiomycota | Tremellomycetes | Trichosporonales | Trichosporonaceae | Apiotrichum | Apiotrichum dehoogii | 0.76 |
| Invaded | f799a4e13333739efddfed364765268b | Fungi | Ascomycota | Archaeorhizomycetes | Archaeorhizomycetales | Archaeorhizomycetaceae | Archaeorhizomyces | Archaeorhizomyces sp | 0.99 |
| Invaded | be90ccd41d48b863f87087d4aa08ff9d | Fungi | Basidiomycota | Agaricomycetes | Polyporales | Ganodermataceae | Ganoderma | NA | 0.98 |
| Invaded | 2db3a4c9d2ad86389ee9d49baff7662a | Fungi | Ascomycota | Sordariomycetes | Hypocreales | Nectriaceae | Dactylonectria | Dactylonectria anthuriicola | 1.00 |
| Invaded | ac35b0cb8561b769c55e16435f8aafe0 | Fungi | Ascomycota | Dothideomycetes | Pleosporales | Melanommataceae | Pleotrichocladium | Pleotrichocladium opacum | 0.94 |
| Invaded | 83e0b558de8048b66a79f7097758fd66 | Fungi | Ascomycota | Sordariomycetes | Amphisphaeriales | Pestalotiopsidaceae | Pestalotiopsis | Pestalotiopsis neglecta | 0.76 |
| Invaded | 17c929ec64bfe11114d6406f3c21131f | Fungi | Basidiomycota | Tremellomycetes | Filobasidiales | Piskurozymaceae | Solicoccozyma | Solicoccozyma aeria | 0.82 |
| Invaded | 5d920f6b7f938aa87946b78fedfe6dc6 | Fungi | Ascomycota | Sordariomycetes | Chaetosphaeriales | Chaetosphaeriaceae | Pseudophialocephala | Pseudophialocephala humicola | 1.00 |
| Invaded | a2cdd08e866697c14bbd00445985369a | Fungi | Rozellomycota | Rozellomycotina cls Incertae sedis | GS05 | GS05 fam Incertae sedis | GS05 gen Incertae sedis | GS05 sp | 0.95 |
| Invaded | 0b6e7bfb448349fde94be2d2ed6986f2 | Fungi | Ascomycota | Sordariomycetes | Hypocreales | Nectriaceae | Thelonectria | Thelonectria olida | 0.82 |
| Rhizosphere | 89c589e18239553d989e1f6ed8f4ee9a | Bacteria | Proteobacteria | Alphaproteobacteria | Rhizobiales A 504705 | Xanthobacteraceae 503485 | Bradyrhizobium | NA | 0.92 |
| Rhizosphere | e8820a8b0ccb14b286019c21e8bfcf00 | Bacteria | Firmicutes D | Bacilli | Bacillales B 310392 | Bacillaceae G 310392 | Bacillus A | NA | 1.00 |
| Rhizosphere | f5818ca485145fe0e2de8d808d811499 | Bacteria | Proteobacteria | Gammaproteobacteria | Burkholderiales 597441 | SHXO01 | SHXO01 | NA | 0.99 |
| Rhizosphere | 9e19be3efeb471bc05e6818a949eb529 | Bacteria | Actinobacteriota | Actinomycetia | Actinomycetales | Cellulomonadaceae | Cellulomonas | NA | 0.99 |
| Rhizosphere | 906b2567ddda9890572015419c397fd6 | Bacteria | Proteobacteria | Gammaproteobacteria | Burkholderiales 597441 | SHXO01 | SHXO01 | NA | 0.98 |
| Rhizosphere | 84b0a2a8482e5f8986477120b857aa77 | Bacteria | Actinobacteriota | NA | NA | NA | NA | NA | 0.99 |
| Rhizosphere | 30f1fe30bb6fa44028182b7ecba785ce | Bacteria | Actinobacteriota | Acidimicrobiia 401430 | Acidimicrobiales | Ilumatobacteraceae | NA | NA | 0.76 |
| Rhizosphere | ba4d475c9d5296670d3625f11aa91404 | Bacteria | Actinobacteriota | Acidimicrobiia 401430 | Acidimicrobiales | Ilumatobacteraceae | NA | NA | 0.74 |
| Rhizosphere | 48d4da2a8f1ff95bc3fb74cff4015710 | Bacteria | Proteobacteria | Gammaproteobacteria | NA | NA | NA | NA | 1.00 |
| Rhizosphere | 06c954688f1e95e67118fb7df5eb1ac1 | Bacteria | Proteobacteria | Gammaproteobacteria | Burkholderiales 597441 | SHXO01 | SHXO01 | NA | 0.73 |
| Rhizosphere | 6fb3367ca906cf78db48909229605734 | Bacteria | Myxococcota A 473307 | Polyangia 463783 | Polyangiales | NA | NA | NA | 1.00 |
| Rhizosphere | e6de82d1d3e1d57633ed7c4c233b6e13 | Bacteria | Actinobacteriota | Acidimicrobiia 401430 | IMCC26256 | IMCC26256 | SHUZ01 | SHUZ01 sp009694415 | 0.78 |
| Rhizosphere | fc4a5d7e90512d7424fb2e939f0d79dd | Fungi | Basidiomycota | Tremellomycetes | Filobasidiales | Piskurozymaceae | Solicoccozyma | Solicoccozyma terricola | 1.00 |
| Rhizosphere | 33adac43d1106225f6f44a857f3b681b | Fungi | Ascomycota | Sordariomycetes | Hypocreales | Nectriaceae | Ilyonectria | Ilyonectria mors-panacis | 0.97 |
| Rhizosphere | 7371fb25c49ccb78a47f5891aa85a994 | Fungi | Ascomycota | Sordariomycetes | Sordariales | Sordariales fam Incertae sedis | Staphylotrichum | Staphylotrichum coccosporum | 1.00 |
| Rhizosphere | c91b38d350962b22a24ad4beb5481d87 | Fungi | Ascomycota | Sordariomycetes | Xylariales | Microdochiaceae | Idriella | Idriella lunata | 0.84 |
| Rhizosphere | 44b248d57966ef3159c6d80c7e9d9424 | Fungi | Ascomycota | Sordariomycetes | Hypocreales | Bionectriaceae | Lasionectriopsis | Lasionectriopsis spinosa | 1.00 |
| Rhizosphere | 4360403cd3e59f847933844e89565fb8 | Fungi | Ascomycota | Sordariomycetes | Hypocreales | Bionectriaceae | Clonostachys | NA | 0.98 |
| Rhizosphere | 4740ac8556d9433dff14c37d15ca1fda | Fungi | Ascomycota | Sordariomycetes | Chaetosphaeriales | Chaetosphaeriaceae | Chloridium | Chloridium aseptatum | 1.00 |
| Rhizosphere | 24e74d80a14c45a5cf5d90ec5a781cc7 | Fungi | Ascomycota | Sordariomycetes | Hypocreales | Nectriaceae | Fusarium | Fusarium solani | 0.75 |
| Rhizosphere | 60ee4f97b4ac5a0cd3c1d6f24b02547f | Fungi | Basidiomycota | Tremellomycetes | Tremellales | Tremellaceae | Tremella | Tremella sp | 1.00 |
| Rhizosphere | bf5b3d89020b717ed5a86a0ad6f1c568 | Fungi | Ascomycota | Dothideomycetes | Pleosporales | Didymellaceae | Epicoccum | Epicoccum sp | 0.72 |
| Rhizosphere | 58900c49fa4c5ed9f183c928cf5802ac | Fungi | Mortierellomycota | Mortierellomycetes | Mortierellales | NA | NA | NA | 0.99 |
| Rhizosphere | ecfed4ed448c412970e5dfeef71342ff | Fungi | Ascomycota | Dothideomycetes | Dothideales | Saccotheciaceae | Aureobasidium | Aureobasidium pullulans | 0.96 |
| Rhizosphere | 6ce7604e712f963bbd0ae0f6b7a3cf5e | Fungi | Ascomycota | Dothideomycetes | Pleosporales | Pleosporaceae | Alternaria | NA | 1.00 |
| Rhizosphere | 8fdf1555fe4887638b1a348f334ab22d | Fungi | Ascomycota | Eurotiomycetes | Eurotiales | Aspergillaceae | Penicillium | Penicillium thomii | 1.00 |
| Rhizosphere | 83e0b558de8048b66a79f7097758fd66 | Fungi | Ascomycota | Sordariomycetes | Amphisphaeriales | Pestalotiopsidaceae | Pestalotiopsis | Pestalotiopsis neglecta | 0.76 |
| Rhizosphere | 6b76917ce6a56b45e66e3b535f7e425d | Fungi | Ascomycota | Leotiomycetes | Helotiales | Helotiales fam Incertae sedis | Mycoarthris | Mycoarthris corallina | 1.00 |
| Rhizosphere | afd50e3dcb2948aa4393b6892fa8a6c2 | Fungi | Ascomycota | Eurotiomycetes | Eurotiales | Aspergillaceae | Penicillium | Penicillium salamorum | 0.80 |
| Rhizosphere | 497bc946b8e334139e9b8aa08ed017f8 | Fungi | Ascomycota | Dothideomycetes | Pleosporales | Amorosiaceae | NA | NA | 0.80 |
| Rhizosphere | 2e4f434bc50d164df90605a9c454d287 | Fungi | Ascomycota | Eurotiomycetes | Eurotiales | Aspergillaceae | Penicillium | Penicillium atrosanguineum | 0.91 |
| Rhizosphere | badcca36ba69a3f4730add597b4004af | Fungi | Ascomycota | Sordariomycetes | Xylariales | Amphisphaeriaceae | Pestalotia | Pestalotia pauciseta | 0.72 |
| Rhizosphere | 31710f04c1d3def7bfea4ec1a43757f0 | Fungi | Ascomycota | Dothideomycetes | Capnodiales | Cladosporiaceae | NA | NA | 1.00 |
| Rhizosphere | ba694f9cfd1867bef35160aa019dd628 | Fungi | Ascomycota | Dothideomycetes | Pleosporales | Phaeosphaeriaceae | Neosetophoma | Neosetophoma poaceicola | 0.88 |
| Rhizosphere | 55e23716cdee8051ed5211cc0cb4ae4a | Fungi | Ascomycota | Sordariomycetes | Phomatosporales | Phomatosporaceae | Phomatospora | Phomatospora sp | 0.71 |
| Rhizosphere | a2cdd08e866697c14bbd00445985369a | Fungi | Rozellomycota | Rozellomycotina cls Incertae sedis | GS05 | GS05 fam Incertae sedis | GS05 gen Incertae sedis | GS05 sp | 0.95 |
| Rhizosphere | 42ed4d417550285aaeed3ab4564755aa | Fungi | Ascomycota | Sordariomycetes | Hypocreales | Nectriaceae | Fusarium | Fusarium culmorum | 0.79 |
| Rhizosphere | d6cc3ad74b741bc8c7cccabc0bf2cafa | Fungi | Ascomycota | Dothideomycetes | Pleosporales | NA | NA | NA | 0.86 |
| Rhizosphere | 20a263126acce408db7a77b214d973ee | Fungi | Ascomycota | Leotiomycetes | Helotiales | NA | NA | NA | 0.93 |
| Rhizosphere | 06fe039e6c439c062fea71deda5adb84 | Fungi | Ascomycota | Dothideomycetes | Pleosporales | Didymellaceae | NA | NA | 1.00 |
| Rhizosphere | 191e424623d0bcab9b26622ecfd5ea8d | Fungi | Ascomycota | Eurotiomycetes | Chaetothyriales | Trichomeriaceae | Trichomerium | Trichomerium cicatricatum | 0.79 |
| Rhizosphere | 15e3e14c27f11faf61566dd5c66256a1 | Fungi | NA | NA | NA | NA | NA | NA | 1.00 |
| Rhizosphere | 173a4c3850b8fe86e33737a877b4f6c1 | Fungi | Ascomycota | Eurotiomycetes | Chaetothyriales | Strelitzianaceae | Strelitziana | NA | 1.00 |
| Rhizosphere | d3c2512e6487148fc8ee3eeef6b985e6 | Fungi | NA | NA | NA | NA | NA | NA | 1.00 |

**Supplemental Figure 1. Rarefaction curve and point estimates for observed amplicon sequences.**

Rarefaction curves were constructed from 10 replicate, random sub-samples taken at each of 10 evenly spaced read depths ranging from 1 to 20,000 reads for both 16S (a) and ITS2 (b) amplicons.


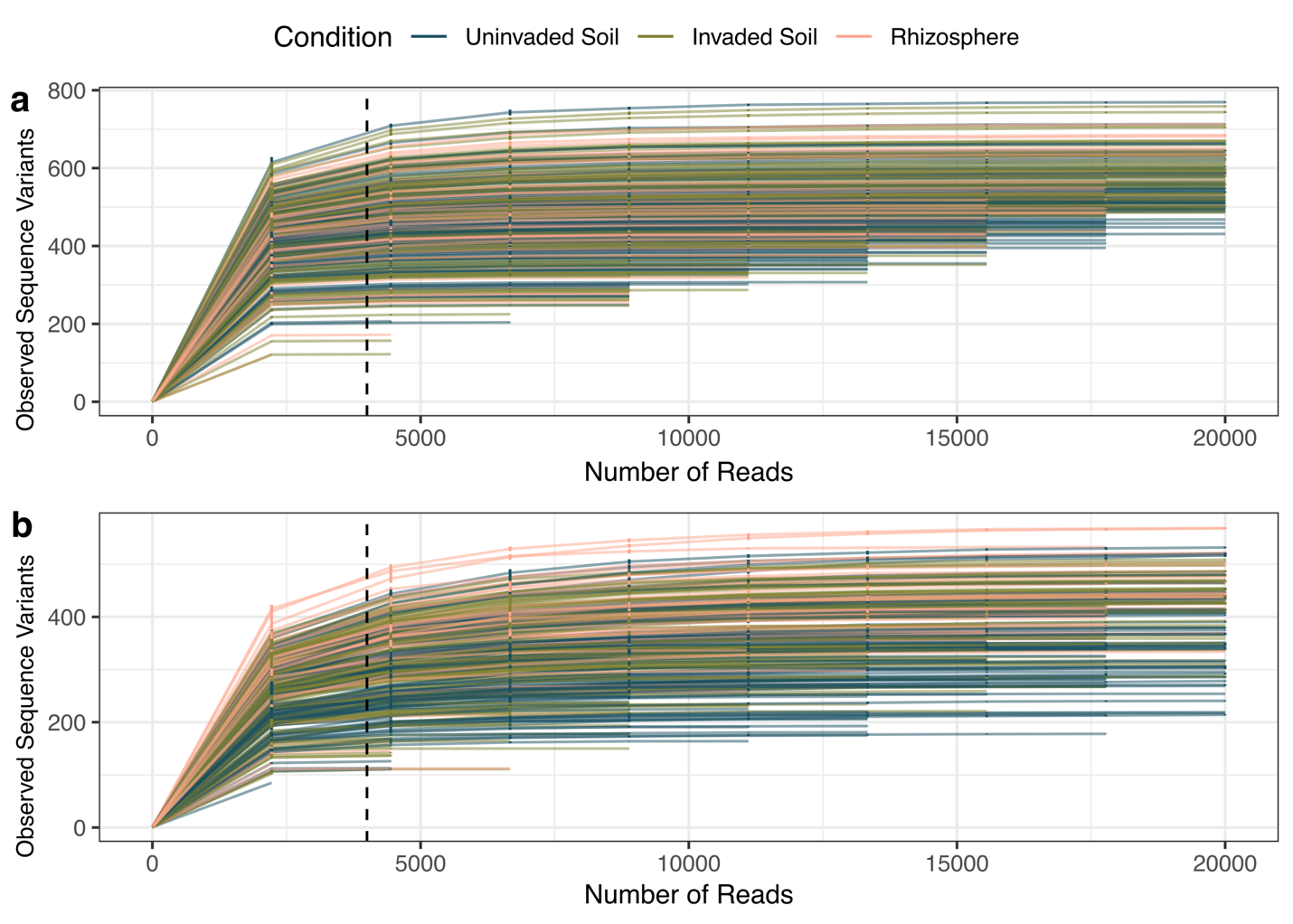

Supplement: Supplemental tables and figure — ANOVA outputs, putative core taxa, and rarefaction curves. [file msphere.00895-25-s0001.docx]
